# Ecological niche modelling as a tool to identify candidate indigenous chicken ecotypes of Tigray (Ethiopia)

**DOI:** 10.3389/fgene.2022.968961

**Published:** 2022-09-30

**Authors:** Gebreslassie Gebru, Gurja Belay, Adriana Vallejo-Trujillo, Tadelle Dessie, Almas Gheyas, Olivier Hanotte

**Affiliations:** ^1^ Tigray Agricultural Research Institute, Mekelle, Ethiopia; ^2^ Addis Ababa University, College of Natural and Computational Science, Department of Microbial, Cellular and Molecular Biology, Addis Ababa, Ethiopia; ^3^ International Livestock Research Institute (ILRI), Addis Ababa, Ethiopia; ^4^ School of Life Sciences, University of Nottingham, Nottingham, United Kingdom; ^5^ Centre for Tropical Livestock Genetics and Health (CTLGH), the Roslin Institute, University of Edinburgh, Edinburgh, United Kingdom; ^6^ Institute of Aquaculture, University of Stirling, Stirling, United Kingdom

**Keywords:** habitat, MaxEnt, climate, agro-ecology, poultry, Tigray

## Abstract

The Tigray region is an ancient entry route for the domestic chickens into Africa. The oldest African chicken bones were found in this region at Mezber, a pre-Aksumite rural farming settlement. They were dated to around 800–400 BCE. Since then, the farming communities of the region have integrated chicken into their livelihoods. The region is also recognised for its high chicken-to-human population ratio and diverse and complex geography, ranging from 500 to 4,000 m above sea level (m.a.s.l.). More than 15 agro-ecological zones have been described. Following exotic chicken introductions, the proportion of indigenous chicken is now 70% only in the region. It calls for the characterisation of indigenous Tigrayan chicken ecotypes and their habitats. This study reports an Ecological Niche Modelling using MaxEnt to characterise the habitats of 16 indigenous village chicken populations of Tigray. A total of 34 ecological and landscape variables: climatic (22), soil (eight), vegetation, and land cover (four), were included. We applied Principal Component Analysis correlation, and MaxentVariableSelection procedures to select the most contributing and uncorrelated variables. The selected variables were three climatic (bio5 = maximum temperature of the warmest month, bio8 = mean temperature of the wettest quarter, bio13 = precipitation of the wettest month), three vegetation and land cover (grassland, forest land, and cultivated land proportional areas), and one soil (clay content). Following our analysis, we identified four main chicken agro-ecologies defining four candidates indigenous Tigrayan chicken ecotypes. The study provides baseline information for phenotypic and genetic characterisation as well as conservation interventions of indigenous Tigrayan chickens.

## Introduction

The multifaceted benefit of the chicken triggered their human-mediated transport to a wide range of environments, which led them to adapt to different agro-ecologies. The Tigray region is likely one of the first routes for the domestic chicken to Africa, with the earliest osteological evidence of chicken in the continent discovered at the Mezber site in the pre-Aksumite rural farming settlement, dated at least 800–400 BCE (Woldekiros and D’Andrea, 2017). Since then, domestic chicken has been integrated into the livelihood of all communities across Ethiopia. Still, the Tigray region is recognised for its higher chicken-to-human population ratio compared to other Ethiopian regions (e.g., 1.3, 0.9, and 0.5 for Tigray, Amhara, and the Oromia regions, respectively) (CSA, 2017, 2020). Furthermore, it has the highest chicken density per km^2^, with 139, 129, and 72 chickens for the Tigray, Amhara, and Oromia regions, respectively (CSA, 2017; 2020). Tigray also comprises diverse eco-geographic areas, following large altitudinal variations, ranging between 500 and 4,000 m above sea level (m.a.s.l.) (Waterbeheer and Van Gaelen, 2011; Haftom et al., 2019). Haftom et al. (2019) have divided the region into 15 agro-ecological zones based on the region’s traditional elevation-based classification and aridity. Despite such agro-ecological diversity, indigenous Tigrayan chicken populations are still grouped as a single category, indigenous chicken, with no specific ecotypes yet recognised. Describing indigenous chickens as one single group while there is such a diverse environment in the Tigray region is likely inappropriate (Vallejo-Trujillo et al., 2022).

As mentioned above, chicken husbandry is old in the region (Woldekiros and D’Andrea, 2017). Also, indigenous chickens are found across the region, where they represent a major source of income for the farmer communities. The agro-ecologies of the Tigray region are dominantly characterized by lowland (<1,500 m. a.s.l.) and midland (1,500 < altitude <2,500 m. a.s.l.) areas, covering 92% of the region (Beyene et al., 2001). The environment is typically warm, with an annual average temperature of 20°C (Hijmans et al., 2005; Fick and Hijmans, 2017). Environmental constraints and human socio-cultural preferences are believed to have shaped the diversity of the chicken. Birds typically have large appendages (to dissipate heat) and have white or light plumage (to shine sunshine). However, in high predation exposed areas, farmers prefer to select dark color chickens, which supposedly will make them less visible to predators (Terfa et al., 2019). Double comb and large frame cocks are the most preferred for breeding and fetch a high price in the market (Alem and Yayneshet, 2013; Asfaw et al., 2017). Hence, besides the environmental adaptation of the chicken, farmers’ and consumers’ preferences have also contributed to adapting the indigenous chicken to the local chicken production systems.

Indigenous chickens are raised under a scavenging system (free range) by the smallholder farmers with little supplementary feed input (e.g., kitchen waste). Accordingly, environmental challenges (e.g., temperature, diseases, feed, water and predation) have been major and selective factors with indigenous chicken expected to be locally adapted.

This lack of recognition of the adaptive diversity of indigenous chickens associated to relatively low productivity have contributed to the massive introduction of commercial breeds in the region. It follows the objectives of the Ethiopian livestock masterplan to increase chicken meat production by 235% and egg production by 828% (Shapiro et al., 2015, 2017), to meet the expected increased demand of 80% for meat and 356% for egg within the country, by the year 2020. Following these introductions, the proportion of exotic chicken and their crossbred is now higher in the Tigray region (30%) compared to the national average (21%), the Amhara (15%) and Oromia (22%) regions (CSA, 2021). This proportion increased by 67% in the past year (CSA, 2020; 2021), following increased consumer demand for chicken products and by-products. This calls for the rapid characterisation of indigenous Tigrayan chicken and their habitats to guide conservation and breeding improvement initiatives. Studying the habitats and defining the potential chicken ecotypes in the Tigray region will provide insight into where and what to conserve.

Ethiopia is one of the countries that has signed the international Convention on Biological Diversity (CBD) (https://www.cbd.int/), with signatory countries committed to take measures to protect biodiversity and to regularly report on the progress (Mackenzie and Jenkins, 2005). Yet, little effort has been undertaken to conserve and protect the indigenous livestock species in the country, including the Tigray region, with only one chicken improvement and conservation program started so far (Hailu et al., 2021). However, considering the low productivity of the indigenous chickens compared to the exotic breeds and their crossbreds, it may be expected that their population size will reduce considerably in the coming years. Accordingly, they may be considered as endangered.

The Ecological Niche Modelling (ENM) approach has been previously used to understand wild species’ habitat distribution and conservation (Thorn et al., 2009). Different algorithms are available for ENM but a prominent method applies maximum entropy modelling - a machine learning algorithm - in MaxEnt software (Phillips et al., 2006). It has better prediction power than other methods and is increasingly becoming the method of choice for habitat characterisation since its first application in 2004 (Morales et al., 2017). The MaxEnt method has numerous advantages: it requires only presence data, it is applicable for both continuous and categorical data simultaneously, it efficiently predicts optimal probability distribution, it is amenable to analysis and it provides continuous outputs (Phillips et al., 2006). The ENM approach has been applied to different species. For examples Nagaraju et al. (2013) applied ENM to identify suitable habitats and to assess regenerating ability and genetic diversity of the Lam tree *Myristica malabarica*. Bentlage and Shcheglovitova (2012) assessed niche similarity of *Anolis* lizard species, Suárez-Mota et al., 2015 used it for the characterisation and conservation of the habitat of *Dyscritothamnus* and *Loxothysanus* flowering plants. Roubicek et al. (2010) used it to study time frame impact of an environmental variable on plants and insects, and Pitt et al. (2016) used it to predict past potentially suitable habitats of domestic chicken across the world in comparison to the habitats of their wild ancestor, Red Junglefowl (*Gallus gallus*).

In livestock, the application of the ENM approach is still in its infancy, with only a few studies so far, primarily in chicken (Vajana et al., 2018; Lozano-Jaramillo et al., 2019; Gheyas et al., 2021; Kebede et al., 2021; Vallejo-Trujillo et al., 2022). Applying ENM on Ethiopian indigenous chickens, Gheyas et al. (2021) identified six major environmental variables. Then, the author chose the extreme environments to identify signatures of positive selection in the genome of these populations associated with the selected environmental parameters. Kebede et al. (2021) studied Ethiopian environmental gradients and using ENM classified the indigenous Ethiopian chicken’s habitats into three agro-ecologies. They reported significant morphological differences between the chicken populations among these agro-ecologies, supporting them as chicken ecotypes (Kebede et al., 2021). In a recent study (Vallejo-Trujillo et al., 2022), described a framework for delineating chicken ecotypes through a detailed environmental characterisation of the population habitats using ENM, followed by the genomic characterisation of the ecotypes. None of the previous studies have fully characterised the Tigrayan indigenous chicken populations that have been adapted to the region’s complex landscape and diversified agro-ecology. For example (Vallejo-Trujillo et al., 2022), study only included Tigrayan indigenous chicken populations living between 1,295 and 2,312 m. a.s.l.

This study was therefore designed to include Tigrayan chicken populations representing all the altitudinal zones of the Tigrayan regions with the aim to identify candidate Tigrayan chicken ecotypes for further phenotypic and genetic characterisation as well as for guiding conservation interventions. Here we have adapted the ENM protocol described in Vallejo-Trujillo et al. (2022) for idenifying the candidate Tigrayan chicken ecotypes and provide a detailed step-by-step desciption of the protocol.

## Material and methods

### Sampling sites and sample size

The study was carried out in the Tigray regional state of Ethiopia, located 556 km away from the capital city Addis Ababa. This region is laid at 12^0^–150 N and 360 30′- 400,30^0^ E and covers ≈54,000 sq km (Yihdego et al., 2018). It has an estimated population of 5.2 million, of which 77% live in rural areas (CSA, 2017). Agriculture is the mainstay of the people. The altitude range from 500 to 4,000 m. a.s.l. And the agro-ecologies comprised 53% lowland (<1,500 m. a.s.l.), 39% midland (1,500 to 2,300 m. a.s.l) and 8% highland (>2,300 m. a.s.l.) areas (Beyene et al., 2001). The soil, geology, vegetation cover, and topography across this region are diverse, which result in different agro-ecologies (Hadgu et al., 2013). The climate of the region is predominantly categorised as semi-arid. The main rainy season is June to mid-September, whereas the warmest season is from March to May.

**FIGURE 1:**
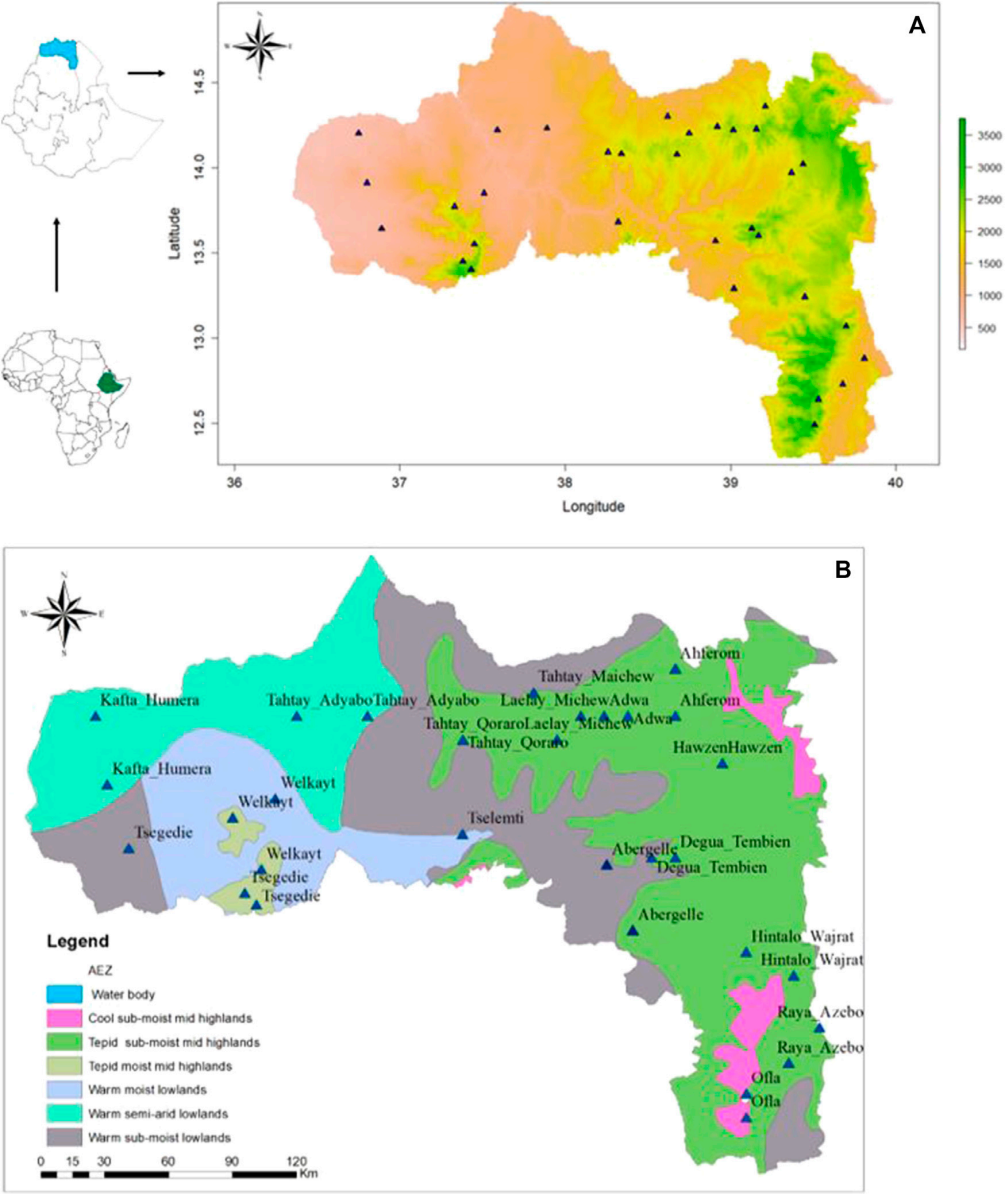
Maps showing the sampling sites of indigenous chickens from the Tigray region **(A)** based on bio-elevation, and **(B)** based on major agro-ecological zones; source (MOA, 1998).

A stratified sampling strategy, based on the agro-ecological zones and the presence of indigenous chicken, (Figure 1B) (Hadgu et al., 2013), was applied to select districts and villages, with a total of 16 districts with 32 villages (Table 1). For each village, latitude and longitude were taken using a geographic positioning system (GPS) GARMIN 72 with an accuracy level of fewer than 3 m (Figure 1A). The district was considered as the entry point for the ENM analysis, so the number of environmental observation for each district depended of the number of villages with 10 data points per village. It ranged from 20 (two villages) to 30 (three villages) data points (Table 1).

**TABLE 1:** Sampling areas.

Wereda (districts)	Villages	Latitude	Longitude	Altitude in meter (m.a.s.l.)	Agroecology
Abergelle	Adi_Weyane	13.55	38.94	1,699	Midland
Abergelle	Lemlem	13.28	39.06	1,598	Lowland
Hawzien	Debrebzien	14.16	39.39	2,187	Midland
Hawzien	Debrehiwot	13.96	39.38	2,106	Midland
Ofla	Selam-Bkalsi	12.65	39.38	2,809	Highland
Raya_Azebo	Genete	12.76	39.68	1,671	Midland
Raya_Azebo	Rabia-Tsigea	12.84	39.63	1924	Midland
Hintalo_wajirat	Mesano	13.24	39.44	2033	Midland
Hintalo_wajirat	Meseret	13.75	39.72	2,158	Midland
Tahtay-Adyabo	Gemhalo	14.57	37.76	1,062	Lowland
Tahtay-Adyabo	May-Kuhli	14.23	37.73	1,111	Lowland
Tahtay-Qoraro	Adi_Gidad	14.09	38.26	1888	Midland
Tahtay-Qoraro	May-Tafat	14.23	38.34	1895	Midland
Kafta_Humera	May-Kadra	14.07	36.56	626	Lowland
Kafta_Humera	Adi-Goshu	14.15	37.35	1,158	Lowland
Kafta_Humera	Adebay	14.20	36.75	665	Lowland
Welkayt	Mogue	14.05	37.49	907	Lowland
Welkayt	Adi_Remets	13.77	37.33	1970	Midland
Tselemti	May_Dagusha	13.68	38.68	1,245	Lowland
Tselemti	Dima	13.68	38.32	1,613	Lowland
Tsegedie	Enda_mariam	13.39	37.41	2,850	Highland
Tsegedie	Enda_Slassie	13.42	37.39	2,584	Highland
Ahferom	Sefo	14.36	39.25	2,175	Highland
Ahferom	May_Keyah	14.42	39.4	2,419	Highland
Adwa	Mariam_Shewito	14.23	39.05	2,227	Highland
Adwa	Bete_Yohannes	14.24	38.92	2,145	Highland
Laelay_Maichew	Dura	14.2	38.75	1943	Midland
Laelay_Maichew	Madego	14.26	38.71	1,662	Lowland
Tahtay_Maichew	Chila	14.3	38.62	1,570	Lowland
Tahtay_Maichew	Shenako	13.83	38.64	2064	Midland
Degua_Tembien	Melfa	13.64	39.13	2,495	Highland
Degua_Tembien	Seret	13.6	39.17	2,494	Highland

### Generating environmental predictors

A total of 34 ecological and landscape variables (Table 2), including 22 climatic, eight soil, and four vegetation and landcover, were selected, based on their biological relevance to indigenous chicken husbandry and suitability for abiotic area classification (Préau et al., 2018). The gridded climatic data (mean values for years 1970–2000) were obtained from the WorldClim database (http://www.worldclim.org/for the variables bio1 - bio19, ‘water vapor pressure’ and elevation) with a spatial resolution of 1 km^2^ (Hijmans et al., 2005; Fick and Hijmans, 2017). The seven soil variables that potentially determine food availability for foraging chickens were obtained from the SoilGrids 1 km v 0.5.8 database (containing global gridded soil information) (Hengl et al., 2014). The water capacity of the soil (mm water per 1 m soil depth) with a 0.5-degree grid was obtained from the Spatial Data Access Tool (SDAT; ORNL DAAC 2017) from NASA (Batjes, 2000). Vegetation and land cover variables (total cultivated land, forest land, and grass/scrub/woodland) that affect both food availability and predation were generated from the ‘Harmonized World Soil Dataset - Land Use and Land Cover’ with a 30 arc-second raster’s (FAO/IIASA/ISRIC/ISS-CAS/JRC, 2009) (Fischer et al., 2008). The crop dominance data were accessed from the Global Food Security Analysis-Support Data (Teluguntla et al., 2015; Oliphant et al., 2017).

**TABLE 2:** Climatic variables analysed.

	Variables	Description	Units	Database
Climate variable	Bio1	Annual Mean Temperature	^0^C	WorldClim - Global Climate Data http://www.worldclim.org/
	Bio2	Mean Diurnal Range (Mean of monthly max temp - mean of monthly min temp)	^0^C(Bio2/Bio7)	
	Bio3	Isothermality (BIO2/BIO7) (* 100)	^0^C	
	Bio4	Temperature Seasonality (standard deviation *100)	^0^C	
	Bio5	Max Temperature of Warmest Month	^0^C	
	Bio6	Min Temperature of Coldest Month	^0^C	
	Bio7	Temperature Annual Range (BIO5-BIO6)	^0^C(Bio5-Bio6)	
	Bio8	Mean Temperature of Wettest Quarter	^0^C	
	Bio9	Mean Temperature of Driest Quarter	^0^C	
	Bio10	Mean Temperature of Warmest Quarter	^0^C	
	Bio11	Mean Temperature of Coldest Quarter	^0^C	
	Bio12	Annual Precipitation	mm/m^2^	
	Bio13	Precipitation of Wettest Month	mm/m^2^	
	Bio14	Precipitation of Driest Month	mm/m^2^	
	Bio15	Precipitation Seasonality (Coefficient of Variation)	mm/m^2^ (Coefficient of variation)	
	Bio16	Precipitation of Wettest Quarter	mm/m^2^	
	Bio17	Precipitation of Driest Quarter	mm/m^2^	
	Bio18	Precipitation of Warmest Quarter	mm/m^2^	
	Bio19	Precipitation of Coldest Quarter	mm/m^2^	
	WatVapPress01	Water Vapor Pressure of the wettest month	kPa	
	WatVapPress01	Water Vapor Pressure of the driest month	kPa	
	Elevation	Meters above sea level	m.a.s.l	
Soil variable	Soil_pH	Soil pH	pH (x10 in H_2_O)	Global gridded soil information https://soilgrids.org/
	Soil_CatEx_Capacity	Soil Cation Exchange Capacity	cmole/kg at depth 0.00 m	
	Soil_Bulk_D	Soil Bulk Density	kg/m3 at depth 0.00 m	
	Soil_Organic Carbon	Soil Organic Carbon	g/kg at depth 0.00 m	
	Soil_Clay	Soil Clay Content	mass fraction in % at depth 0.00 m	
	Soil_Silt	Soil Silt Content	mass fraction in % at depth 0.00 m	
	Soil_Sand	Soil Sand Content	mass fraction in % at depth 0.00 m	
	Soil_Water_Capacity	Soil total available Water Capacity	mm2/1 mt soil depth	Spatial Data Access Tool (SDAT)-NASA https://webmap.ornl.gov/ogc/dataset.jsp?ds_id=546
Vegetation variable	Forest	Forest Cover	%	Harmonized World Soil Dataset http://www.fao.org/soils-portal/soil-survey/soil-maps-and-databases/harmonized-world-soil-database-v12/en/
	Grass_Land	Grass/Shrub Land	%	
	Cult_L	Land use for agricultural purpose (Cultivated land)	%	
	Crop_Dominance	Crop Dominance (major crops)	Category	Global Food Security Analysis-Support DATA https://croplands.org/

R packages’ rgdal’, ‘maptools’, ‘rgeos’, and ‘raster’ were used to adjust the dimension and extension of the grid to 1 km^2^ based on the earth-fixed terrestrial reference system and geodetic datum WGS84 for the agro-ecological variable ‘raster’ layers. Rather than representing the sampling area with a single point, we added proximate areas to enhance the probability of an accurate description of the area. Hence, each sampling population represents ten sampling points (one sampling point and nine nearby surrounding areas within 1.2 km^2^ taken as random mid-point sample). Google Earth Pro 7.3.1.4507 (2016) was applied, to identify these points, and then the separate grids were extracted using the ‘raster’ R package.

### Environmental variable selection procedure

The environmental variables were analysed with two investigative approaches—Spearman correlation and Principal Component Analysis (PCA), and one selection process - ‘MaxentVariableSelection’ (MVS). First, we examined the correlations among the environmental variables. The strengths of correlation was defined as: r_s_ ≥ 0.8 very strong, r_s_ ≥ 0.6 to <0.8 strong, r_s_ ≥ 0.4 to <0.6 moderate, r_s_ ≥ 0.2 to <0.4 weak, and r_s_ ≥ 0 to <0.2 very weak. Since most of the environmental variables (except soil cation exchange) did not follow a normal distribution pattern (Shapiro-Wilk normality test output: W = 0.6–0.98; *p* = 2.20e-16 to 4.60e-04), correlations among the variables were tested using Spearman’s rank correlation coefficients (r). The correlations were evaluated using a threshold value of 0.6 with *p* < 0.0001. The results were plotted using the R package ‘corrplot’ (Wei et al., 2017). Then, the PCA was performed to assess each variable’s contribution and relationship in their respective group (climatic, soil, and vegetation and land cover) using the R package ‘stats’. The variables’ eigenvector (direction) and eigenvalue (magnitude) were assessed in the PCA-based inspection. In the final variable selection process, all variables were simultaneously evaluated using the R package MVS (Jueterbock, 2015) to select a set of uncorrelated and high contributing variables for the execution of the ENM.

### Selection of model parameters

The performance of ENM can be affected by the model parameters—feature class (FC) and beta-multiplier (BM), and the default setting may not be ideal for generating maximum entropy (Anderson and Gonzalez, 2011; Cao et al., 2013). The FC work on transforming the environmental predictors to model complex relationships (Elith et al., 2010). The BM helps to prevent over-complexity or overfitting of the model by manipulating the intensity of the nominated FC (Merow et al., 2013). ENMeval package (Kass et al., 2021) was applied to choose the best combination of FC and BM. The following FCs were tested: linear (L), quadratic (Q), product (*p*), hinge (H), categorical (C), and threshold (T), in combination with different values of BM ranging from one to tweleve. The least Akaike Information Criterion, corrected for small samples (AICc) values, was considered as the point for the optimal FC and BM combination (Muscarella et al., 2014). The best FC and BM identified here were used to optimise the MaxEnt and to develop the suitability maps.

### Ecological niche modelling

MaxEnt (ver. 3.4.1; https://biodiversityinformatics.amnh.org/open_source/maxent/) was used for the ENM analysis of the environments of district (Phillips et al., 2006). First, we ran the MaxEnt with all 320 points as a single entity to evaluate the model’s performance, and then we run it for the individual 16 districts. In each ENM run, we withheld 25% of the occurrence data as testing, while we used the remaining 75% as training; we then applied the regionalised ten k-fold cross-validations (Vallejo-Trujillo et al., 2022). We used the logarithmic scale of logistic and cumulative outputs to develop the niches. The logistic output was used for the pairwise comparison of the models and the cumulative one was used to display the suitability habitat.

We used the Area Under the Receiver Operating Characteristics (ROC) Curve (AUC) to evaluate the model’s accuracy (Phillips et al., 2006). The AUC values ranged from 0 to 1, where 1 is most suitable, and 0 is unsuitable, with values in between showing suitability in gradient. Further, the jackknife was used to assess the importance and contribution of the variables.

### Pairwise comparison of population models for ecotypes definition

The ENMTools Perl software (Warren et al. (2008); Warren and Seifert, (2011)) was applied to assess the similarity of habitats among the populations following Warren et al. (2008) approach that runs in two steps. In the first procedure, we run Pearson’s pairwise correlation between the population models, with coefficient values ranging from -1 to +1 (negative values indicate negative linear correlation, positive values indicate positive linear relationship, and if it approaches zero, it indicates no linear correlation between the district’s niches). The second procedure used Hellinger’s distance or Niche overlap (noted with the letter ‘I’) with values ranging from 0 to 1 (a value close to one means the niches are closely similar, and a value close to zero, they are entirely distinct) (Warren et al., 2008). Similarity metrices from these two methods were then used to cluster the populations into candidate ecotypes as follows.

We applied ‘stats’ and ‘cluster’ packages of R to calculate the “Euclidean distance” of each dataset (Pearson’s pairwise correlation and Hellinger’s distance). Using these Euclidean distance values, we performed hierarchical clustering. We clustered the topologies by calculating the agglomerative coefficients of the single or minimum linkage, complete or maximum linkage, average or UPGMA, and Ward methods. We chose the Ward method due to its largest agglomerative coefficient value that explains the strength of the structuring.

**FIGURE 2:**
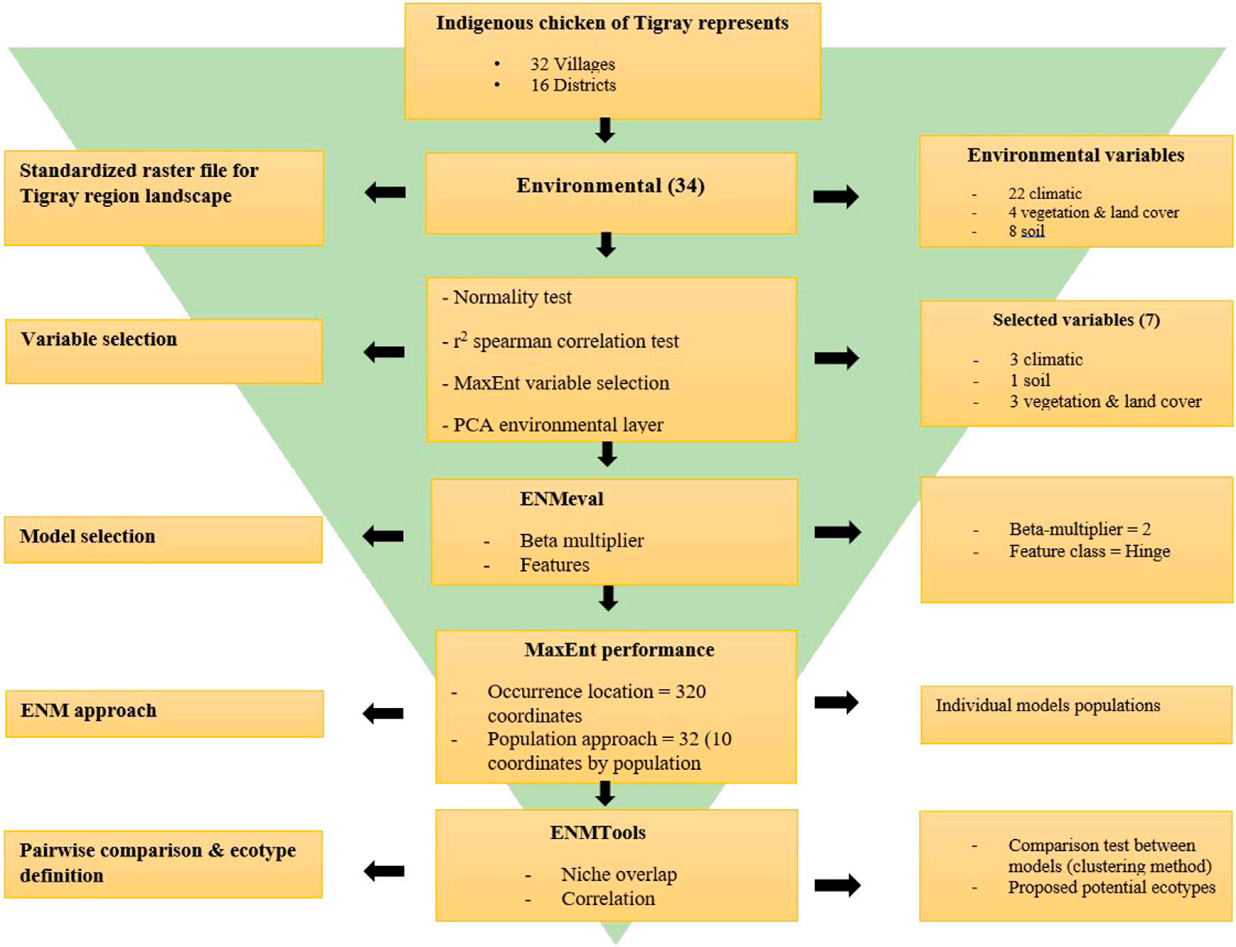
Flowchart of pipeline for Tigrayan chicken ecotype definition following the Ecological Niche Modelling (ENM) approach.

Finally, for easy visualisation of the similarity between ENMs, we generated dendrograms and heatmaps for each dataset using the ‘ggplot2’ and ‘Reshape2’ R packages. This helped to classify the populations from the districts into potential ecotypes in combination with the Jackknife of AUC and percent contribution of the variables. The summary of the procedure we followed is presented in Figure 2.

## Results

### Correlation-based explanation of variables

**FIGURE 3:**
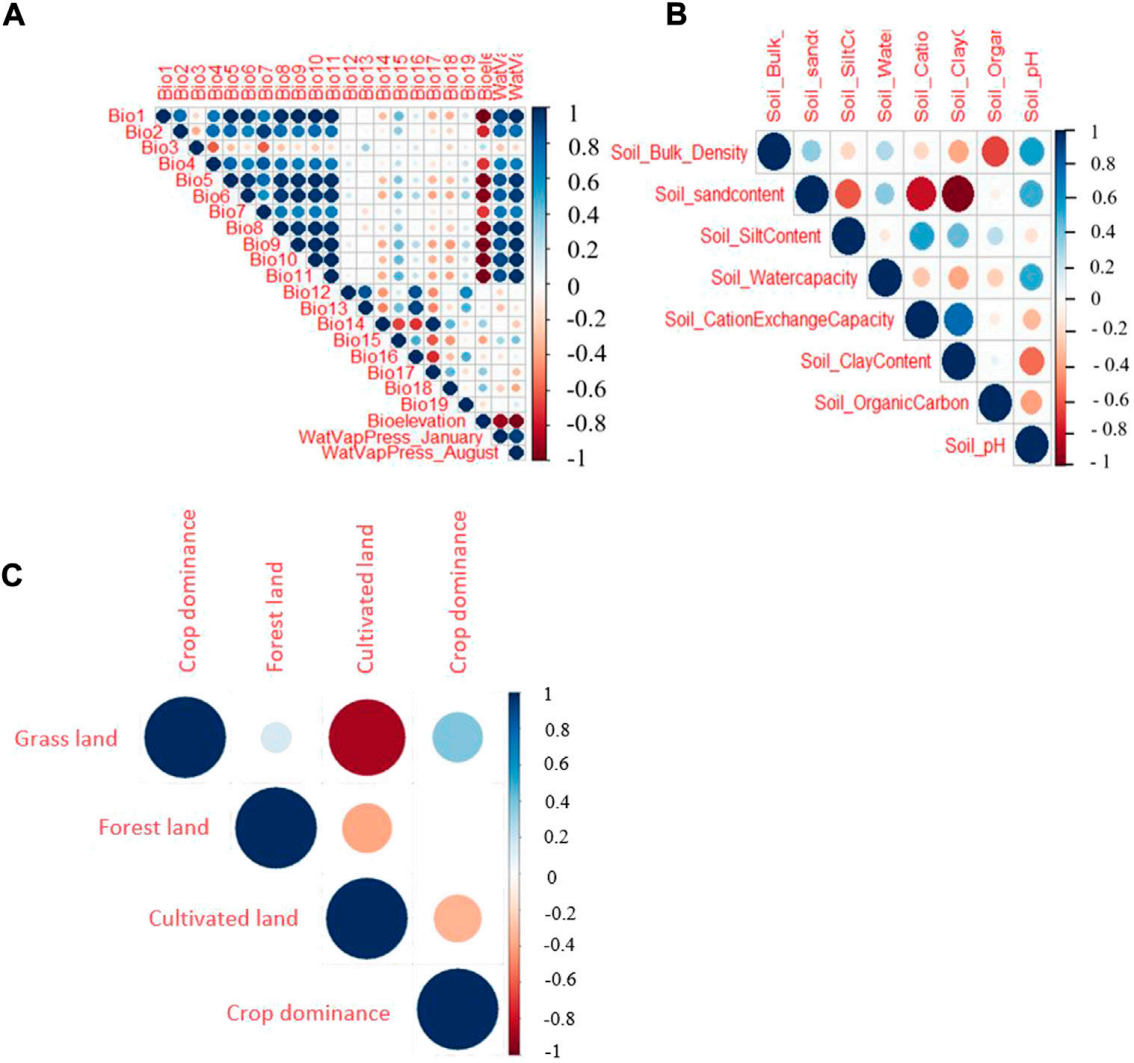
Spearman correlation test for three different groups of climatic and environmental parameters evaluated **(A)** Climatic variables, **(B)** soil variables, **(C)** vegetation and land cover variables.

Before using for ENM, the environmental variables were shortlisted by removing highly correlated variables and those with a low contribution to the model. Hence, a threshold of r_s_ > 0.6 (with a *p*-value of 0.001) was used to remove variables from a correlated set except for the one variable expected to be the most relevant one to the indigenous chicken biology. The Spearman’s rank-order correlation results for the three groups of variables (climatic ‘A’, soil ‘B’, and vegetation and land cover ‘C’) are shown in Figure 3. In the soil group, there are five variables with a correlation coefficient <0.6 (the threshold for retaining variables for ENM). These are the pH, water capacity, and contents of organic matter, clay, and silt. The vegetation and land cover group (forest land, crop dominance, and grassland) are not strongly correlated. Based on the correlation from the climatic variables, we find six variables with a correlation <0.6 out of the 22 variables, namely bio3 (isothermality), bio10 (mean temperature of the warmest quarter), bio13 (precipitation of the wettest month), bio17 (precipitation of the driest quarter), bio18 (precipitation of the warmest quarter), and bio19 (precipitation of the coldest quarter).

### PCA of Tigrayan chicken populations based on agro-climatic variables

**FIGURE 4:**
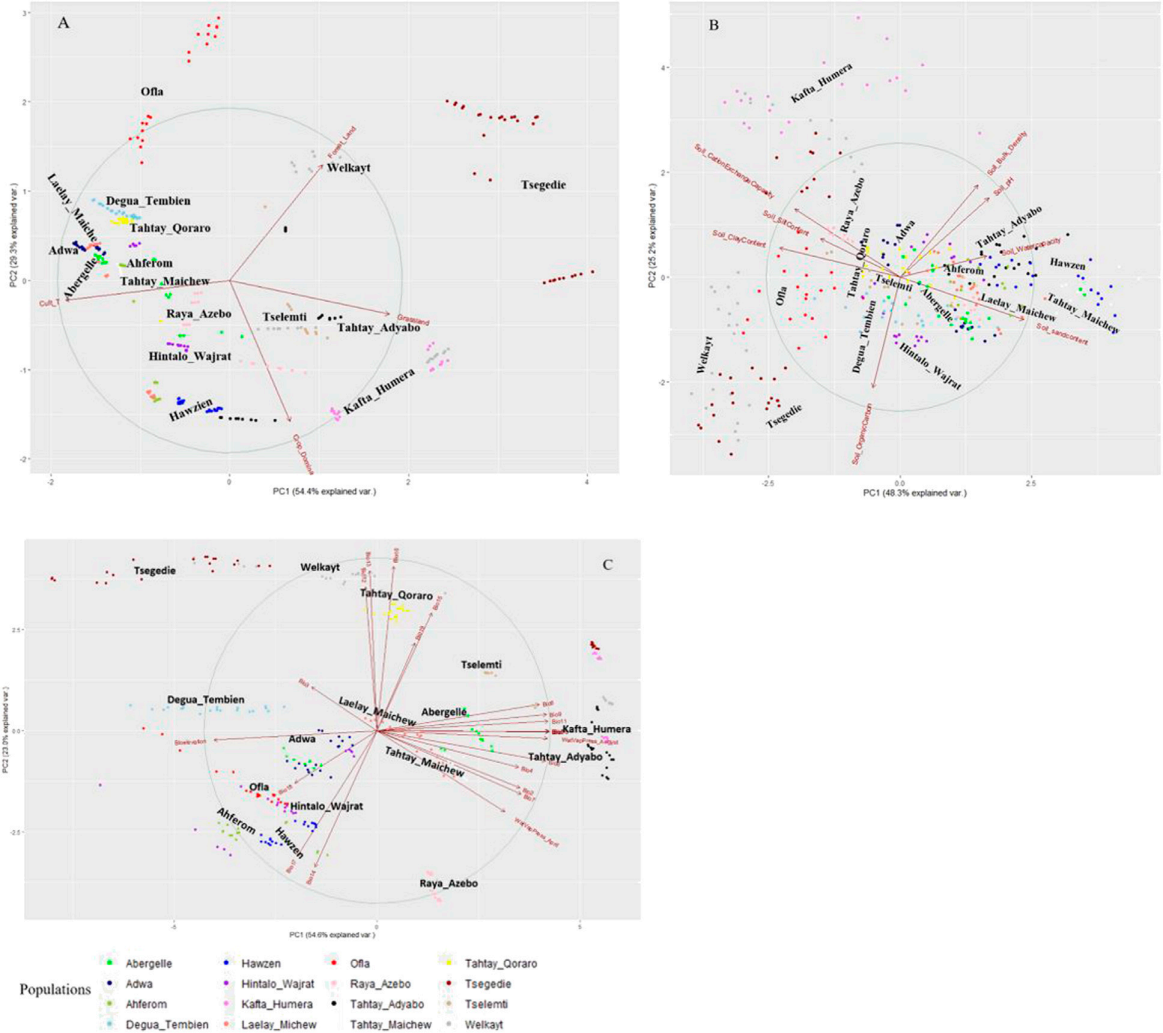
Principal component analyses of 16 Tigray chicken districts based on agro-ecological and climatic variables **(A)** vegetation and land cover variables, **(B)** soil variables, and **(C)** climatic variables.

We perform the PCA-based clustering to see how the 34 environmental variables cluster the indigenous chicken population and the association of each variable with the population. The PCA plots of Tigrayan chicken samples based on environmental data (Figure 4) helped us to assess the environmental variables’ contribution and association. The arrows and direction of the variables show which PC and axis (x or y) are associated with the shown variance. The arrow lengths indicate the extent of the contribution of a variable in explaining the populations’ environmental structure. The first two principal components (PC1 and PC2) represent more than 73% of the variance, with the distribution of the populations widely different among the PCAs using the three defined variable clusters (vegetation and landcover ‘A', Soil ‘B', and climatic ‘C'). All four vegetation and land cover variables show high variation (Figure 4A). For the soil variable, we see several variables showing contribution in the same direction (no variation) (e.g. soil bulk density and soil pH, or soil clay content and soil cation exchange capacity) (Figure 4B). Four variables, namely soil organic, sand, clay, and water capacity content, show high variation. Within the climatic variables (Figure 4C), the water vapor pressure for the hottest month (April) and bio-elevation show strong variation and no association with other variables. On the contrary, variables bio3, bio4, bio18, and bio19 show weak contributions. Despite differences between PC plots, several districts appeared repeatedly close to each other, such as Welkayt - Tsegedie, Degua Tembien—Tahtay Qoraro, Tselemti—Tahtay Adyabo, and Adwa—Laelay Maichew. It suggests a homogeneous landscape configuration for these districts.

### MaxentVariableSelection (MVS) package for variable selection

**FIGURE 5:**
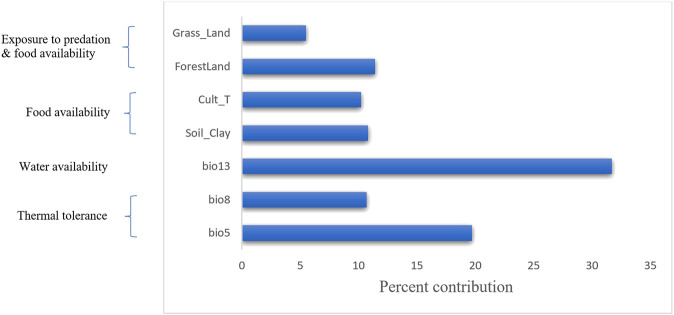
Percent contribution of the final selected seven variables using MVS. The left-hand side of the diagram shows the biological importance of these variables to environmental adaptation in chicken.

While the correlation and principal component analyses described above explored the relationships among variables (soil, vegetation and land cover, and climatic), MVS was used to simultaneously analyse all variables to select the most important set of uncorrelated variables (r < 0.6). Accordingly, seven variables (three from climatic variables (bio5 = maximum temperature of the warmest month, bio8 = mean temperature of the wettest quarter, and bio13 = precipitation of the wettest month), three from vegetation and land cover variables (grassland, forestland, and cultivated land) and one soil variable (clay content)) were selected (Figure 5).

### Selection of model parameters

**FIGURE 6:**
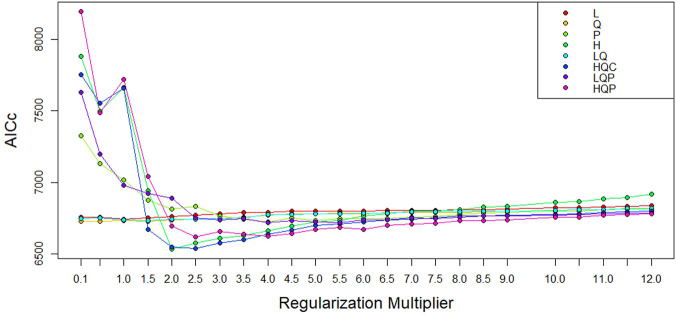
AICc values for analyzed feature class (FC) combinations using different beta-multiplier (BM) values using ENMeval.

To develop the optimal ENM, we selected the best combination of FC (H) and BM (=2) out of the192 model combinations evaluated using the ENMeval package (Figure 6, Supplementary Table S1). The selected FC and BM combination resulted in a better prediction potential of the suitable niches than the default values (Supplementary Figure S1).

### ENM development using selected predictors and parameters

**FIGURE 7:**
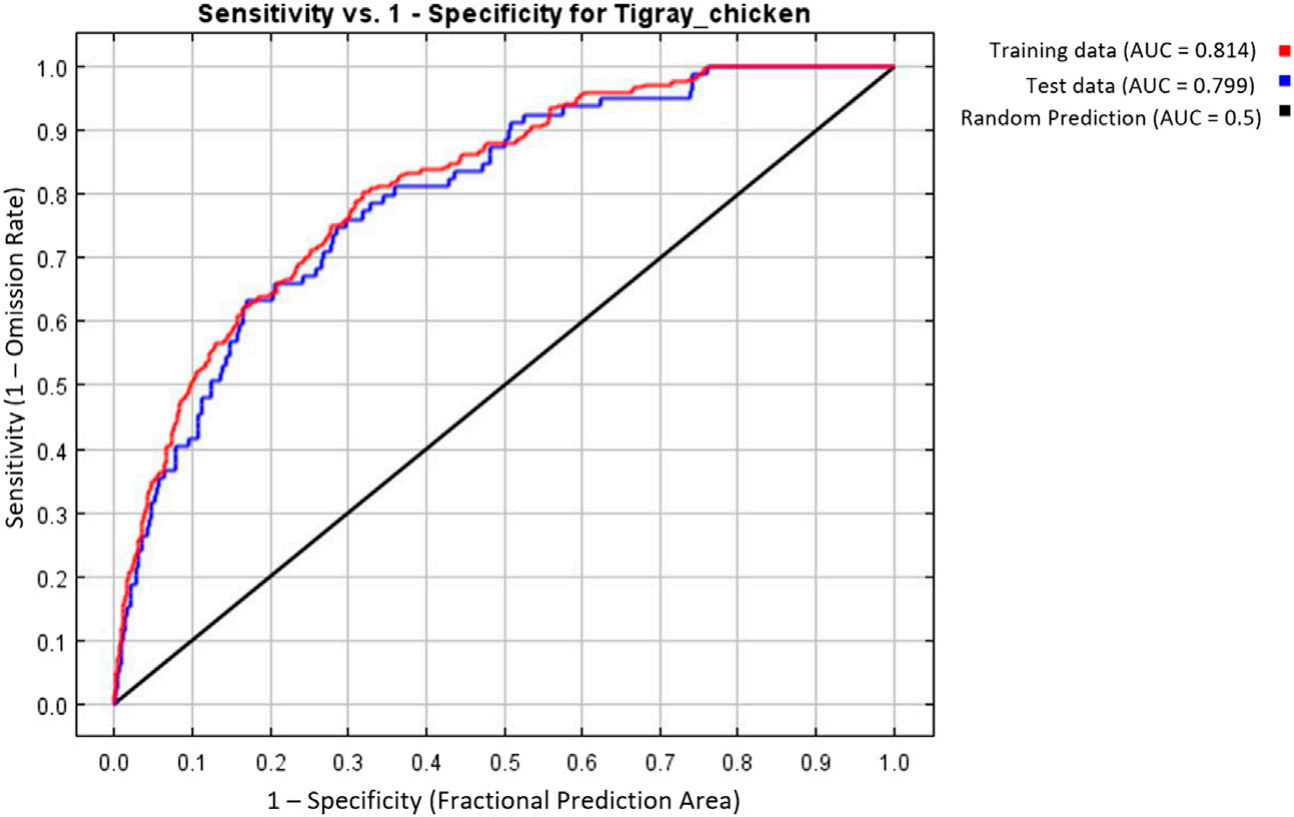
MaxEnt model based on seven selected variables and the best feature (H) and beta-multiplier value (two). Area Under Receiver Operating Curve for training and test data.

**FIGURE 8:**
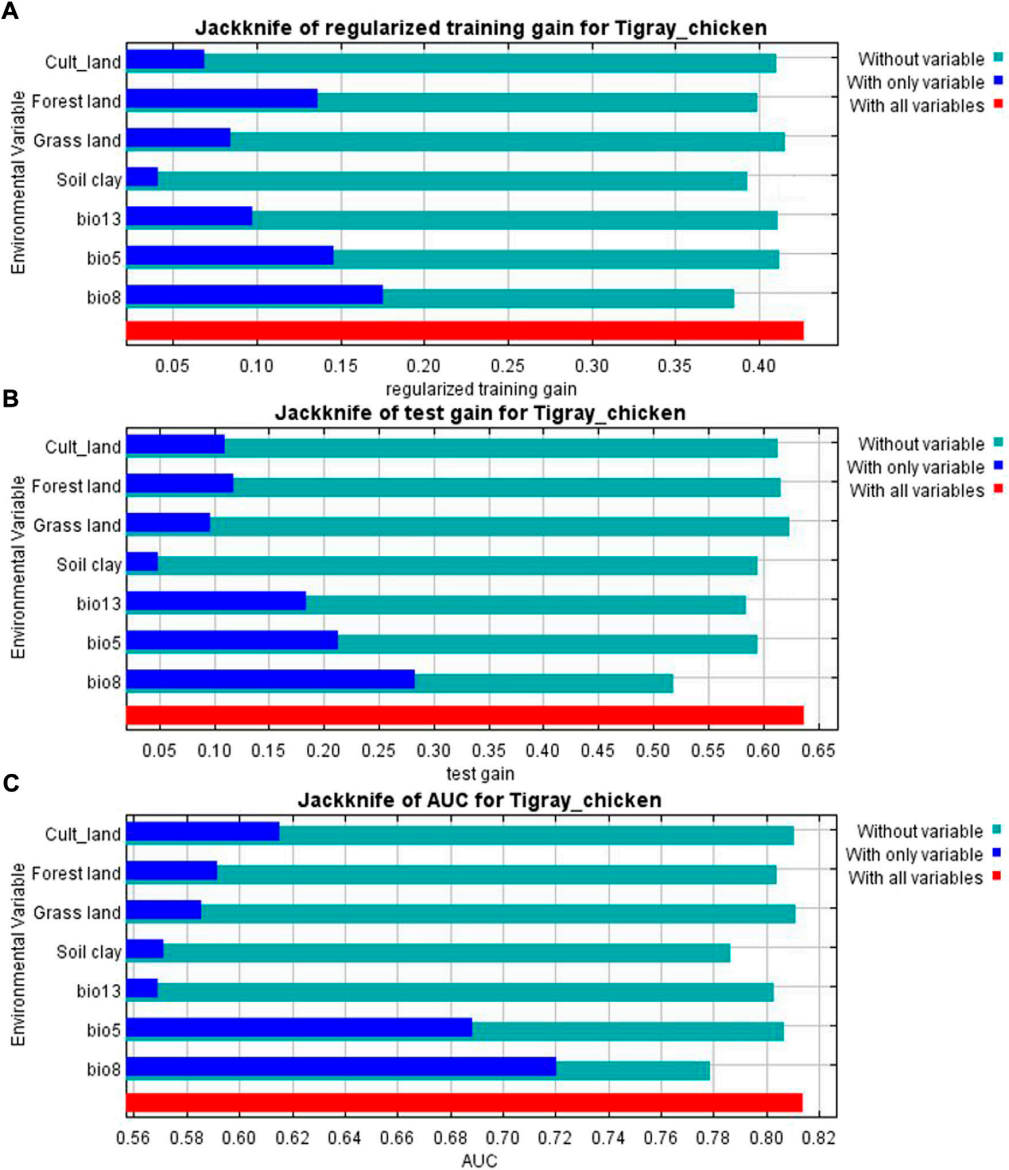
**(A)** Jackknife result for AUC (Area Under Receiver Operating Curve) **(B)** Jackknife of training gain and **(C)** test gain for ENM produced for the complete set of analyzed populations.

**FIGURE 9:**
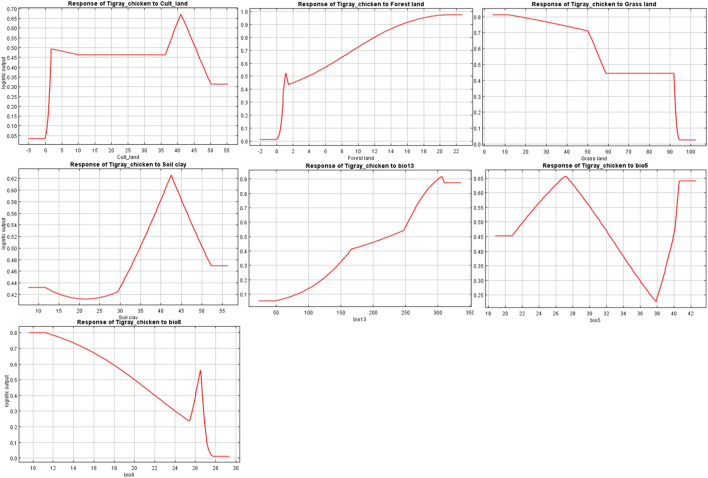
Individual response curves for seven environmental variables selected for the final Maxent model.

MaxEnt model was executed with the seven selected variables and the best combination of FC (Hinge) and BM (=2) to predict the suitability habitat of each indigenous chicken population and to identify environmental variables that define each habitat. To evaluate the model prediction efficiency, MaxEnt produces different outputs (Figures 7–9). The AUC values 0.814 and 0.799 for training and test data, respectively (Figure 7), showed the model’s excellent prediction power. The AUC values of the individual populations range from 0.9854 to 0.9981 for the training sample and from 0.9693 to 0.9946 for the test sample. The individual variable AUC value also displayed excellent prediction power (>0.9) except for a few variables across different populations showing moderate prediction power (Supplementary Table S3).

The variables’ importance was also checked through the jackknife tests (Figures 8A,B) for regularized training and test gains. According to these tests, the variables that contributed most to the model were bio13, bio5, and bio8. Most of the selected variables contributed to the overall model (Figure 8C), except the soil clay content. Moreover, variable importance was not only evaluated by their contribution to the prediction power of the model but also their contribution to the model building (Çoban et al., 2020). Variables that contributed most to the model were also explained by their percentages of contribution (>6.9) and their permutation importance (>9.8 except bio13) (Supplementary Table S2) with the response curves explaining how the individual variables affect ENM prediction. The logistic prediction varied with the individual variable while the other variables were kept at their average value (Figure 9).

### Pairwise comparison of population models for ecotypes definition

**FIGURE 10:**
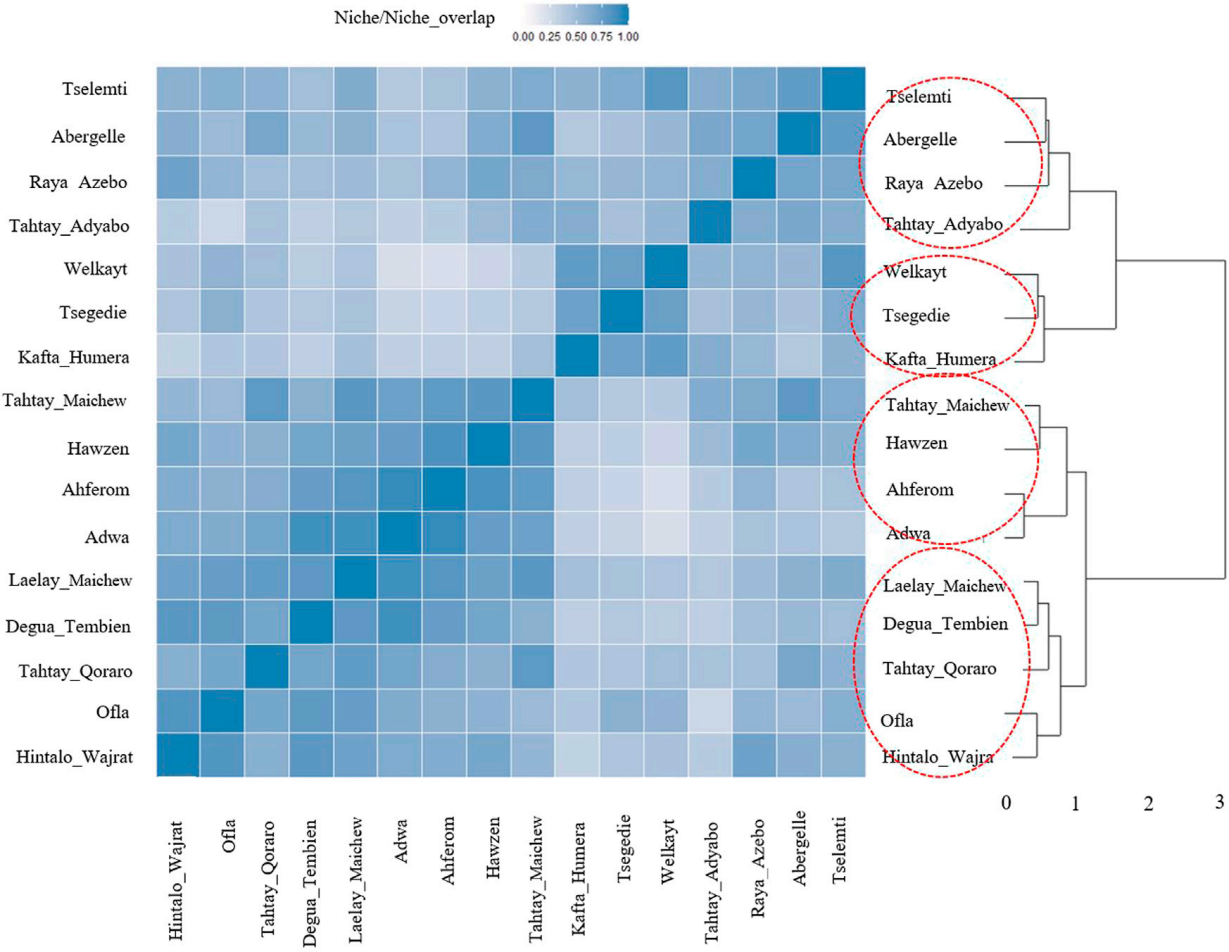
Dendrogram and heatmap of niche overlap statistics (I) between suitability maps for individual population.

**FIGURE 11:**
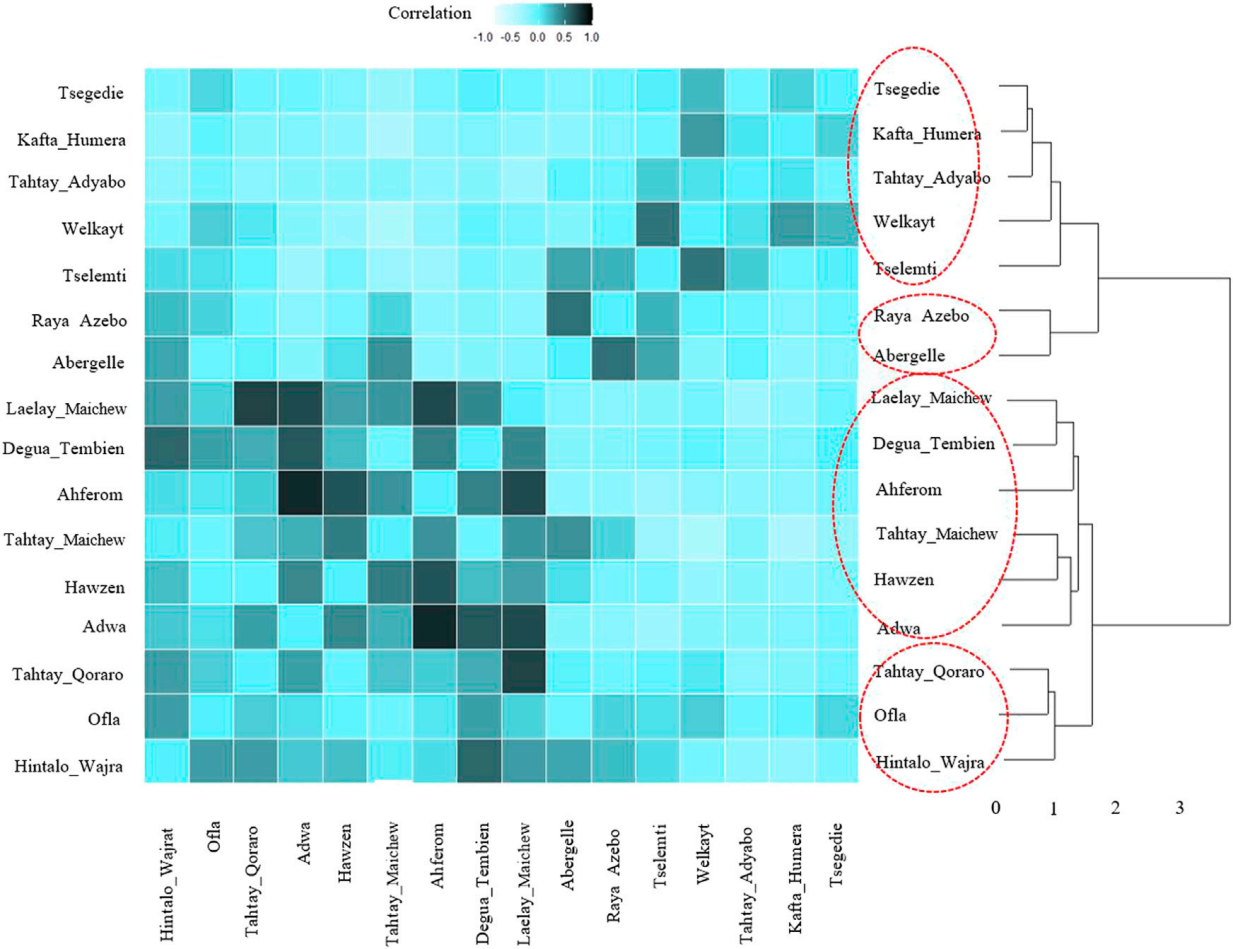
Dendrogram and heatmap of pairwise Pearson correlation coefficient between suitability maps for individual population.

For the ecotype definition, we present two approaches, pairwise Pearson correlation and niche overlap (Vallejo-Trujillo et al., 2022). Both methods group the Tigray indigenous chicken habitats into four agro-ecologies. Twelve districts out of 16 showed consistent clustering in both methods. The exceptions are Tselemti, Tahtay Adyabo, Degua Tembien, and Laelay Maichew. The highest agglomerative coefficient was obtained with Ward Method of hierarchical clustering - 0.83 for niche overlap and 0.77 for the Pearson correlation (Figures 10, 11). Henceforth, it was selected for the clustering of the Tigray chicken populations.

**FIGURE 12:**
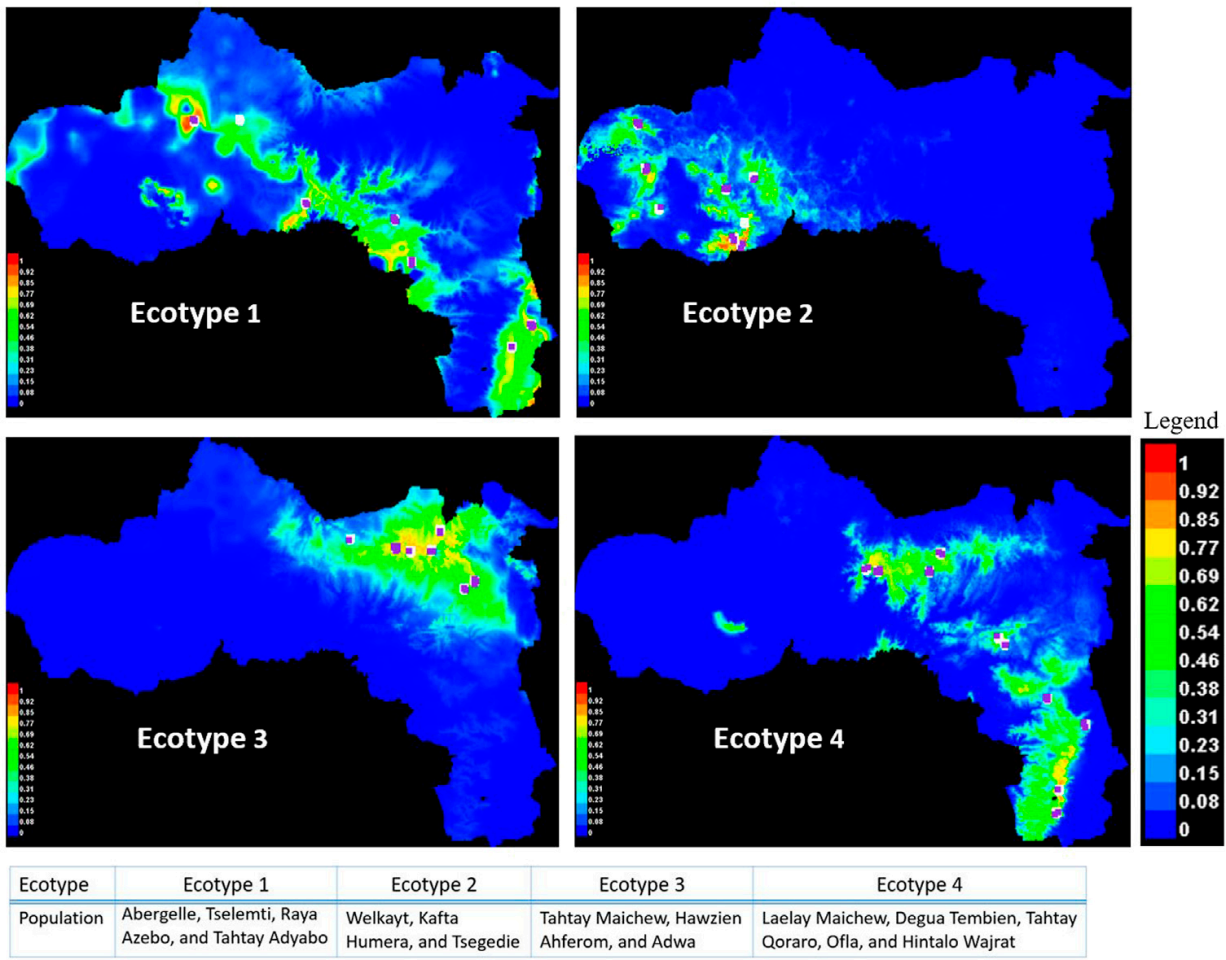
Suitability maps for Tigray chicken populations grouped by ecotype.

Besides the above dendrogram and heat map categories, by considering their Jackknife of AUC and percent of the contribution, we proposed four agro-ecologies, namely: agro-ecology 1 - Tselemti, Abergelle, Tahtay Adyabo, and Raya Azebo; agro-ecology 2 - Welkayt, Tsegedie, and Kafta Humera; agro-ecology 3 - Tahtay Maichew, Hawzen, Ahferom, and Adwa; and agro-ecology 4 - Tahtay Maichew, Degua Tembien, Tahtay Qoraro, Ofla, and Hintalo Wajrat. We also identified the environmental variables that define each suitable agro-ecology (Table 3). Following the four distinct chicken agro-ecologies, we proposed four distinct indigenous chicken ecotypes (Figure 12).

**TABLE 3:** Major contributing agro-ecological variables for each proposed ecotype.

Proposed ecotypes	Populations	Major contributor parameters among ecotypes (percent of contribution)
1	Abergelle, Tselemti, Raya Azebo, and Tahtay Adyabo	bio5 (39%), Forest (21.4%), Grass_Land (18.8), and Cult_L (17.2%)
2	Welkayt, Kafta Humera, and Tsegedie	bio13 (41.9%), Cult_L (26.4%), Soil_Clay (15.9%), bio5 (9%), bio8 (5.1%)
3	Tahtay Maichew, Hawzien Ahferom, and Adwa	bio5 (50.4%), Forest (40.5%), and Cult_L (8.2%)
4	Laelay Maichew, Degua Tembien, Tahtay Qoraro, Ofla, and Hintalo Wajrat	bio8 (43.4%), Grass_Land (24.8%), Soil_Clay (17.4%), and Cult_L (5.1%)

Bio5 = Maximum temperature of warmest month; bio8 = Mean temperature of the wettest quarter; bio13 = Precipitation of wettest month; Forest = Forest cover; Soil_Clay = Soil clay content; Cult_L = land use for agriculture purpose; Grass_Land = Grass/shrub cover.

## Discussion

We have applied here an ecological niche modelling approach, using 34 agro-climatic variables. It allowed us to provide the first detailed environmental characterisation of Tigrayan chicken habitats, an important premise to define ecotypes and to study their adaptive diversity, with important implications for their management, conservation, phenotypic and genetic characterisation. Besides, we have also provided here the detailed protocols for the application of ENM, that we expect will facilitate its adoption for the environmental characterization of livestock population habitats.

Although, some studies have already been undertaken to define chicken agro-ecological zones in Ethiopia, they did not represent (Kebede et al., 2021) or poorly represented (Gheyas et al., 2021; Vallejo-Trujillo et al., 2022) the Tigray region. Specifically, the studies of Gheyas et al. (2021) and Vallejo-Trujillo et al. (2022) did not include any population >2,312 m. a.s.l. And below 1,295 m. a.s.l.

### Environmental variable selection and their contribution to chicken biology

ENM for suitable habitat prediction and potential ecotype definition require selecting appropriate variables to enhance the model prediction power. Prior knowledge of the species ecology is also essential in selecting the correct environmental variables (Zeng et al., 2016; Fourcade et al., 2018). Besides, the variables must be related to the life history of the species under study. Previous studies have emphasized that the variables for ENM must be selected with great care as they should be uncorrelated and with a high contribution to the biological need of the species (Reunanen, 2003; Zhu et al., 2012; Sangermano et al., 2015; Pitt et al., 2016). Removing correlated variables using PCA, correlation matrix, or any other dimension reduction methods will reduce model complexity (Merow et al., 2013). Hence, we followed a rigorous procedure, including PCA, correlation, and MVS, to select the least correlated variables. The procedures followed and our first-hand knowledge of the study area and of indigenous chicken helped us to select the most appropriate variables.

The final set of selected variables are indeed of relevance to the physiological need of the chicken; bio5 (maximum temperature of the warmest month) and bio8 (mean temperature of the wettest quarter) may be associated with bird thermotolerance - an important phenotype in the Tigray region where the temperature may fluctuate between 3 and 46°C. Bio13 (precipitation of the wettest month) is linked to water availability, equally crucial for the physiology of the chicken (Pitt et al., 2016). Cultivated land and soil clay content are associated with food availability, and grassland and forest land coverage may be related to scavenging food, predation exposure and protection (Figure 5) (Gheyas et al., 2021; Vallejo-Trujillo et al., 2022). Our selected variables (grassland cover, forest cover, cultivated land, and soil clay content) aligned with the variables (grassland cover, cultivated land, crop dominance, and soil organic content) reported previously in other studies for Ethiopian indigenous chicken (Gheyas et al., 2021; Vallejo-Trujillo et al., 2022).

The shortlisted climatic variables bio5, bio8, and bio13 based on MVS are also related to the climatic variables bio6 (minimum temperature of coldest month), bio15 (precipitation seasonality), bio16 (precipitation of wettest quarter), and bio17 (precipitation of driest quarter) shortlisted in previous studies (Gheyas et al., 2021; Vallejo-Trujillo et al., 2022). The variables selected in our study are also similar to variables (bio5, bio13, soil clay, grassland, forest land, and cultivated land) reported for the wild Red junglefowl (Pitt et al., 2016). Pitt et al. (2016) reported geographic areas suitable to domestic chicken on the African continent using the environmental information associated to today’s geographic distribution of the Red Junglefowl. The Tigray region is one of these regions. Tigray is also a center of ancient civilization which is geographically close to Red Sea and ancient commercial maritime routes. Interestingly, it is in the Tigray region that the early osteological evidence of domestic chicken have been found on the African continent (Woldekiros and D’Andrea, 2017).

### Ecological niche modelling procedures for agro-ecology classification and ecotype definition

Running MaxEnt with the default “black-box” does not guarantee an optimal model (Radosavljevic and Anderson, 2014). Instead, it may produce either over-complex or over-simple models Phillips et al. (2006); Cao et al. (2013); Elith et al. (2010); Ribeiro et al. (2016); Shcheglovitova and Anderson (2013), and overall suitability niche output can vary when applied with default and optimum model parameters, and attention should be given to the methodology when using MaxEnt for ENM (Morales et al., 2017). Hence, to get quality MaxEnt outputs, we need to be cautious in selecting the two parameters, feature classes and regularisation multiplier (Warren and Seifert, 2011; Merow et al., 2013; Morales et al., 2017). While selecting the two parameters, we should also consider the region’s geographic boundaries (Merow et al., 2013).

We applied the ENMeval package to choose the best-fit settings, hinge (H) FC and BM = 2 (Figure 6) to predict suitable habitats for the studied areas. The selected H feature uses a linear function in the fitted function to transform the continuous predictors (environmental variables) to a binary output, zero below the threshold and one above the threshold (Elith et al., 2011). Using the H feature class in model development has numerous advantages: it produces smooth models, it allows complex relationships to be modelled in training data, it contributes to model improvement, it is considered as default by MaxEnt, it is applicable to a small number of sampling sites (minimum 15), and it may replace the quadratic product, and threshold features (Phillips and Dudı, 2008; Elith et al., 2010; Merow et al., 2013). The BM (2) we chose also helped us control the model complexity by imposing a penalty.

Validation of the developed niches using statistical tests boosts the biological meaning of the model (Warren and Seifert, 2011). Therefore, we evaluate the niche similarities of the indigenous chicken populations using ENMtools by calculating the niche similarity and correlation between suitability maps (dendrogram and heat map). Besides the validation using ENMtools, we considered the Jackknife AUC and percent of contribution to further confirm the proposed four agro-ecologies.

As expected, geographically close districts generally clustered together into the same ENM agro-ecology; e.g., agro-ecology 1 Tselemti and Abergelle, agro-ecology 4 Tahtay Qoraro, and Laelay Maichew, and agro-ecology 2 and 3 for the other districts (Figure 12). However, some districts belong to different agro-ecologies despite being geographically close (Ofla and Raya Azebo; Ahferom, Adwa, and Laelay Maichew). The Ofla district belong to agro-ecology 4 and the Raya Azebo district to agro-ecology 1. Similarly, the Ahferom, Adwa, and Laelay Maichew district while geographically close displayed minimum niche overlap. They are classified in different agro-ecologies with Ahferom and Adwa district included within agro-ecology 1, and the Laelay Maichew district within agro-ecology 2. It illustrates the diversity of agro-ecologies found within the Tigray region, even within a close geographic range.

The occurrence of new habitats due to environmental change is the main reason for the formation of a “new variety” (Darwin, 2004) or, in this current context “ecotype”. Darwin also emphasised that populations that adapt to a new environment may survive, resulting in the gradual formation of new species. Transpose to the evolution of livestock, introducing a population to a novel habitat may result in new phenotypes following natural selection (Schluter and Nagel, 1995; Adams and Huntingford, 2004; Lahti et al., 2009). Accordingly, we propose four potential Tigrayan chicken ecotypes that may display different chicken genotypes and phenotypes.

The identification of these four ecotypes may further guide both genetic improvement and conservation efforts, maintaining the unique adaptation of the indigenous populations. Different strategies may be envisaged here. Within ecotypes productivity improvement may be envisaged at poultry stations, as it has been the case in Ethiopia for the Horro chicken (Dana et al., 2011), or following an open nucleus breeding scheme *in-situ* at village level (Gondwe et al., 2001; Okeno et al., 2013). Alternatively, crossbreeding may be envisaged, for example, crossing of improved local cocks lines with a commercial hen (Kgwatalala et al., 2015). The later may results in immediate productivity improvement, while keeping local environmental adaptation. A medium to long term, it will be important to conserve the unique environmental characteristics of the ecotypes protecting them, for examples, from the local impact of extreme climatic events or political instability (e.g., war). It is now possible to conserve *ex-situ* in biobank male and female poultry primordial germ cells (Hu et al., 2022). Ecotype identification will allow prioritizing the populations to be conserved by providing entry points for the establishment of poultry biobanks.

## Conclusion

The environment-based characterization of chicken habitats presented here allowed us to propose four potential indigenous chicken ecotypes associated with different environmental variables. Beyond the current objective, the outcome of this study will guide the conservation of the endangered indigenous chicken ecotypes while providing a standardised framework for new studies on the environmental characterisation of livestock populations, and its link to phenotypes and genotypes. Also, the ecological niche modelling approach describe here may be used to predict future environmental challenges and distributional shift that indigenous chicken ecotypes may be facing owing to climate changes.

## Data Availability

The original contributions presented in the study are included in the article/Supplementary Material, further inquiries can be directed to the corresponding authors.
