# Supplementary material for: Ecological niche modelling as a tool to identify candidate indigenous chicken ecotypes of Tigray (Ethiopia)
Source: Front Genet. 2022 Sep 30;13:968961. doi: 10.3389/fgene.2022.968961 (PMC9561088; doi:10.3389/fgene.2022.968961)
Supplement: Supplementary file 1 [file DataSheet1.docx]

**Supplementary (Tables and Figures) for:** **Ecological niche modelling as a tool for classifying agro-ecologies and identification of indigenous chicken ecotypes of Tigray (Ethiopia)**

**Suppl. Table 1: ENMeval table results for all combination of features and beta multipliers.**

|  | Settings | features | rm | train.AUC | avg.test.AUC | var.test.AUC | avg.diff.AUC | var.diff.AUC | avg.test.orMTP | var.test.orMTP | avg.test.or10pct | var.test.or10pct | AICc | delta.AICc | w.AIC | parameters |
| --- | --- | --- | --- | --- | --- | --- | --- | --- | --- | --- | --- | --- | --- | --- | --- | --- |
| 1 | L_0.1 | L | 0.1 | 0.765 | 0.750 | 0.023 | 0.032 | 0.014 | 0.013 | 0.001 | 0.116 | 0.006 | 6754.3 | 221.2 | 8.27E-49 | 31 |
| 2 | Q_0.1 | Q | 0.1 | 0.779 | 0.763 | 0.022 | 0.033 | 0.012 | 0.009 | 0.001 | 0.125 | 0.004 | 6729.2 | 196.1 | 2.31E-43 | 33 |
| 3 | P_0.1 | P | 0.1 | 0.866 | 0.841 | 0.011 | 0.031 | 0.007 | 0.006 | 0.000 | 0.134 | 0.003 | 7323.7 | 790.6 | 1.86E-172 | 184 |
| 4 | H_0.1 | H | 0.1 | 0.937 | 0.894 | 0.011 | 0.036 | 0.009 | 0.009 | 0.000 | 0.178 | 0.010 | 7879.6 | 1346.5 | 3.56E-293 | 165 |
| 5 | LQ_0.1 | LQ | 0.1 | 0.796 | 0.778 | 0.022 | 0.032 | 0.014 | 0.003 | 0.000 | 0.131 | 0.006 | 6744.7 | 211.6 | 9.96E-47 | 53 |
| 6 | HQC_0.1 | HQC | 0.1 | 0.935 | 0.898 | 0.014 | 0.035 | 0.010 | 0.006 | 0.000 | 0.163 | 0.005 | 7752.0 | 1218.9 | 1.84E-265 | 148 |
| 7 | LQP_0.1 | LQP | 0.1 | 0.874 | 0.850 | 0.010 | 0.027 | 0.008 | 0.009 | 0.000 | 0.131 | 0.005 | 7627.8 | 1094.7 | 1.69E-238 | 208 |
| 8 | HQP_0.1 | HQP | 0.1 | 0.934 | 0.844 | 0.170 | 0.023 | 0.005 | 0.013 | 0.000 | 0.147 | 0.005 | 8194.7 | 1661.6 | 0 | 184 |
| 9 | L_0.5 | L | 0.5 | 0.764 | 0.749 | 0.022 | 0.031 | 0.013 | 0.009 | 0.000 | 0.100 | 0.005 | 6754.1 | 221.0 | 9.16E-49 | 27 |
| 10 | Q_0.5 | Q | 0.5 | 0.773 | 0.759 | 0.023 | 0.032 | 0.014 | 0.009 | 0.001 | 0.116 | 0.003 | 6729.0 | 195.9 | 2.50E-43 | 29 |
| 11 | P_0.5 | P | 0.5 | 0.860 | 0.838 | 0.013 | 0.031 | 0.011 | 0.006 | 0.000 | 0.134 | 0.004 | 7132.0 | 598.9 | 7.65E-131 | 166 |
| 12 | H_0.5 | H | 0.5 | 0.937 | 0.913 | 0.003 | 0.027 | 0.003 | 0.006 | 0.000 | 0.147 | 0.005 | 7498.1 | 965.0 | 2.49E-210 | 129 |
| 13 | LQ_0.5 | LQ | 0.5 | 0.788 | 0.771 | 0.022 | 0.030 | 0.013 | 0.013 | 0.001 | 0.122 | 0.003 | 6751.2 | 218.1 | 3.79E-48 | 49 |
| 14 | HQC_0.5 | HQC | 0.5 | 0.934 | 0.910 | 0.004 | 0.029 | 0.004 | 0.009 | 0.000 | 0.166 | 0.007 | 7553.0 | 1019.9 | 3.02E-222 | 141 |
| 15 | LQP_0.5 | LQP | 0.5 | 0.863 | 0.842 | 0.012 | 0.030 | 0.009 | 0.009 | 0.000 | 0.131 | 0.005 | 7198.4 | 665.3 | 3.00E-145 | 176 |
| 16 | HQP_0.5 | HQP | 0.5 | 0.948 | 0.907 | 0.007 | 0.032 | 0.005 | 0.009 | 0.000 | 0.181 | 0.005 | 7486.3 | 953.2 | 8.99E-208 | 141 |
| 17 | L_1 | L | 1 | 0.761 | 0.745 | 0.023 | 0.031 | 0.012 | 0.009 | 0.001 | 0.103 | 0.005 | 6744.2 | 211.1 | 1.29E-46 | 21 |
| 18 | Q_1 | Q | 1 | 0.769 | 0.756 | 0.022 | 0.031 | 0.013 | 0.009 | 0.001 | 0.113 | 0.003 | 6730.8 | 197.7 | 1.05E-43 | 26 |
| 19 | P_1 | P | 1 | 0.850 | 0.827 | 0.012 | 0.033 | 0.010 | 0.009 | 0.000 | 0.147 | 0.003 | 7018.7 | 485.6 | 3.15E-106 | 139 |
| 20 | H_1 | H | 1 | 0.924 | 0.900 | 0.005 | 0.028 | 0.005 | 0.009 | 0.000 | 0.159 | 0.007 | 7663.7 | 1130.6 | 2.67E-246 | 129 |
| 21 | LQ_1 | LQ | 1 | 0.781 | 0.765 | 0.022 | 0.030 | 0.013 | 0.009 | 0.001 | 0.106 | 0.005 | 6738.3 | 205.2 | 2.40E-45 | 40 |
| 22 | HQC_1 | HQC | 1 | 0.935 | 0.905 | 0.005 | 0.026 | 0.004 | 0.006 | 0.000 | 0.166 | 0.007 | 7658.4 | 1125.3 | 3.84E-245 | 134 |
| 23 | LQP_1 | LQP | 1 | 0.860 | 0.832 | 0.015 | 0.031 | 0.011 | 0.006 | 0.000 | 0.141 | 0.004 | 6979.0 | 445.9 | 1.34E-97 | 146 |
| 24 | HQP_1 | HQP | 1 | 0.937 | 0.912 | 0.004 | 0.027 | 0.003 | 0.013 | 0.000 | 0.172 | 0.006 | 7718.5 | 1185.4 | 3.36E-258 | 154 |
| 25 | L_1.5 | L | 1.5 | 0.754 | 0.741 | 0.022 | 0.031 | 0.012 | 0.009 | 0.001 | 0.103 | 0.005 | 6754.0 | 220.9 | 9.60E-49 | 21 |
| 26 | Q_1.5 | Q | 1.5 | 0.766 | 0.754 | 0.022 | 0.030 | 0.012 | 0.009 | 0.001 | 0.106 | 0.002 | 6729.3 | 196.2 | 2.17E-43 | 22 |
| 27 | P_1.5 | P | 1.5 | 0.847 | 0.819 | 0.014 | 0.032 | 0.010 | 0.003 | 0.000 | 0.131 | 0.004 | 6872.8 | 339.7 | 1.51E-74 | 123 |
| 28 | H_1.5 | H | 1.5 | 0.916 | 0.888 | 0.009 | 0.028 | 0.007 | 0.009 | 0.000 | 0.159 | 0.005 | 6941.5 | 408.4 | 1.79E-89 | 125 |
| 29 | LQ_1.5 | LQ | 1.5 | 0.774 | 0.762 | 0.020 | 0.029 | 0.012 | 0.009 | 0.001 | 0.103 | 0.005 | 6734.8 | 201.7 | 1.37E-44 | 32 |
| 30 | HQC_1.5 | HQC | 1.5 | 0.912 | 0.882 | 0.010 | 0.029 | 0.008 | 0.006 | 0.000 | 0.141 | 0.007 | 6669.7 | 136.6 | 1.93E-30 | 123 |
| 31 | LQP_1.5 | LQP | 1.5 | 0.847 | 0.819 | 0.017 | 0.035 | 0.012 | 0.003 | 0.000 | 0.138 | 0.003 | 6922.8 | 389.7 | 2.05E-85 | 132 |
| 32 | HQP_1.5 | HQP | 1.5 | 0.928 | 0.901 | 0.006 | 0.028 | 0.005 | 0.009 | 0.000 | 0.159 | 0.006 | 7039.7 | 506.7 | 8.42E-111 | 155 |
| 33 | L_2 | L | 2 | 0.751 | 0.736 | 0.022 | 0.032 | 0.012 | 0.009 | 0.001 | 0.103 | 0.005 | 6760.1 | 227.0 | 4.36E-50 | 21 |
| 34 | Q_2 | Q | 2 | 0.763 | 0.751 | 0.022 | 0.030 | 0.012 | 0.009 | 0.001 | 0.103 | 0.003 | 6735.5 | 202.4 | 9.59E-45 | 22 |
| 35 | P_2 | P | 2 | 0.832 | 0.810 | 0.014 | 0.033 | 0.010 | 0.006 | 0.000 | 0.116 | 0.002 | 6813.6 | 280.5 | 1.08E-61 | 106 |
| 36 | H_2 | H | 2 | 0.894 | 0.866 | 0.011 | 0.029 | 0.008 | 0.009 | 0.000 | 0.125 | 0.005 | 6533.1 | 0.0 | 0.877265818 | 93 |
| 37 | LQ_2 | LQ | 2 | 0.771 | 0.756 | 0.021 | 0.030 | 0.013 | 0.009 | 0.001 | 0.103 | 0.005 | 6743.1 | 210.0 | 2.24E-46 | 32 |
| 38 | HQC_2 | HQC | 2 | 0.885 | 0.853 | 0.016 | 0.033 | 0.012 | 0.006 | 0.000 | 0.100 | 0.005 | 6548.8 | 15.7 | 0.000347639 | 95 |
| 39 | LQP_2 | LQP | 2 | 0.831 | 0.812 | 0.019 | 0.033 | 0.011 | 0.006 | 0.000 | 0.122 | 0.002 | 6890.2 | 357.1 | 2.48E-78 | 120 |
| 40 | HQP_2 | HQP | 2 | 0.914 | 0.882 | 0.008 | 0.030 | 0.007 | 0.006 | 0.000 | 0.166 | 0.005 | 6695.2 | 162.1 | 5.53E-36 | 137 |
| 41 | L_2.5 | L | 2.5 | 0.745 | 0.732 | 0.023 | 0.032 | 0.012 | 0.006 | 0.000 | 0.106 | 0.005 | 6771.0 | 237.9 | 1.90E-52 | 21 |
| 42 | Q_2.5 | Q | 2.5 | 0.759 | 0.748 | 0.022 | 0.030 | 0.012 | 0.009 | 0.001 | 0.103 | 0.002 | 6743.4 | 210.3 | 1.85E-46 | 22 |
| 43 | P_2.5 | P | 2.5 | 0.818 | 0.804 | 0.018 | 0.034 | 0.012 | 0.006 | 0.000 | 0.122 | 0.002 | 6830.8 | 297.7 | 1.94E-65 | 102 |
| 44 | H_2.5 | H | 2.5 | 0.877 | 0.848 | 0.013 | 0.031 | 0.010 | 0.009 | 0.000 | 0.116 | 0.005 | 6574.1 | 41.0 | 1.12E-09 | 77 |
| 45 | LQ_2.5 | LQ | 2.5 | 0.768 | 0.755 | 0.021 | 0.030 | 0.012 | 0.009 | 0.001 | 0.106 | 0.005 | 6743.5 | 210.4 | 1.82E-46 | 29 |
| 46 | HQC_2.5 | HQC | 2.5 | 0.860 | 0.834 | 0.018 | 0.033 | 0.012 | 0.006 | 0.000 | 0.103 | 0.005 | 6537.0 | 3.9 | 0.1223865 | 61 |
| 47 | LQP_2.5 | LQP | 2.5 | 0.830 | 0.800 | 0.022 | 0.036 | 0.014 | 0.006 | 0.000 | 0.125 | 0.003 | 6751.1 | 218.0 | 4.03E-48 | 89 |
| 48 | HQP_2.5 | HQP | 2.5 | 0.896 | 0.861 | 0.011 | 0.032 | 0.009 | 0.003 | 0.000 | 0.141 | 0.005 | 6619.0 | 85.9 | 1.93E-19 | 112 |
| 49 | L_3 | L | 3 | 0.741 | 0.728 | 0.023 | 0.032 | 0.012 | 0.006 | 0.000 | 0.109 | 0.004 | 6777.9 | 244.8 | 6.08E-54 | 20 |
| 50 | Q_3 | Q | 3 | 0.756 | 0.744 | 0.021 | 0.030 | 0.012 | 0.009 | 0.001 | 0.100 | 0.003 | 6748.8 | 215.7 | 1.26E-47 | 21 |
| 51 | P_3 | P | 3 | 0.820 | 0.792 | 0.017 | 0.034 | 0.012 | 0.003 | 0.000 | 0.119 | 0.001 | 6766.0 | 232.9 | 2.34E-51 | 88 |
| 52 | H_3 | H | 3 | 0.862 | 0.834 | 0.014 | 0.031 | 0.010 | 0.009 | 0.000 | 0.122 | 0.009 | 6611.5 | 78.4 | 8.13E-18 | 67 |
| 53 | LQ_3 | LQ | 3 | 0.763 | 0.750 | 0.021 | 0.030 | 0.012 | 0.009 | 0.001 | 0.103 | 0.004 | 6748.1 | 215.0 | 1.77E-47 | 27 |
| 54 | HQC_3 | HQC | 3 | 0.845 | 0.822 | 0.019 | 0.033 | 0.012 | 0.013 | 0.000 | 0.116 | 0.006 | 6574.7 | 41.6 | 7.93E-10 | 54 |
| 55 | LQP_3 | LQP | 3 | 0.817 | 0.792 | 0.019 | 0.035 | 0.012 | 0.006 | 0.000 | 0.106 | 0.002 | 6738.3 | 205.2 | 2.48E-45 | 77 |
| 56 | HQP_3 | HQP | 3 | 0.876 | 0.845 | 0.013 | 0.033 | 0.010 | 0.006 | 0.000 | 0.128 | 0.005 | 6655.6 | 122.5 | 2.22E-27 | 105 |
| 57 | L_3.5 | L | 3.5 | 0.735 | 0.723 | 0.024 | 0.031 | 0.012 | 0.006 | 0.000 | 0.109 | 0.004 | 6788.9 | 255.8 | 2.54E-56 | 19 |
| 58 | Q_3.5 | Q | 3.5 | 0.752 | 0.739 | 0.022 | 0.030 | 0.012 | 0.006 | 0.000 | 0.097 | 0.002 | 6758.0 | 224.9 | 1.26E-49 | 21 |
| 59 | P_3.5 | P | 3.5 | 0.805 | 0.782 | 0.017 | 0.034 | 0.012 | 0.009 | 0.000 | 0.113 | 0.002 | 6742.8 | 209.7 | 2.60E-46 | 70 |
| 60 | H_3.5 | H | 3.5 | 0.850 | 0.825 | 0.014 | 0.030 | 0.010 | 0.009 | 0.000 | 0.122 | 0.006 | 6627.6 | 94.5 | 2.67E-21 | 56 |
| 61 | LQ_3.5 | LQ | 3.5 | 0.760 | 0.745 | 0.022 | 0.031 | 0.012 | 0.013 | 0.002 | 0.109 | 0.004 | 6753.7 | 220.6 | 1.10E-48 | 26 |
| 62 | HQC_3.5 | HQC | 3.5 | 0.833 | 0.810 | 0.018 | 0.032 | 0.011 | 0.013 | 0.000 | 0.119 | 0.006 | 6599.8 | 66.7 | 2.82E-15 | 48 |
| 63 | LQP_3.5 | LQP | 3.5 | 0.802 | 0.783 | 0.019 | 0.034 | 0.013 | 0.009 | 0.000 | 0.113 | 0.002 | 6745.5 | 212.4 | 6.53E-47 | 71 |
| 64 | HQP_3.5 | HQP | 3.5 | 0.860 | 0.834 | 0.013 | 0.032 | 0.009 | 0.006 | 0.000 | 0.125 | 0.005 | 6638.6 | 105.5 | 1.08E-23 | 88 |
| 65 | L_4 | L | 4 | 0.732 | 0.721 | 0.024 | 0.031 | 0.012 | 0.003 | 0.000 | 0.106 | 0.003 | 6789.4 | 256.4 | 1.89E-56 | 16 |
| 66 | Q_4 | Q | 4 | 0.747 | 0.734 | 0.022 | 0.030 | 0.012 | 0.006 | 0.000 | 0.103 | 0.002 | 6769.0 | 235.9 | 5.32E-52 | 21 |
| 67 | P_4 | P | 4 | 0.800 | 0.778 | 0.018 | 0.032 | 0.012 | 0.003 | 0.000 | 0.113 | 0.002 | 6724.1 | 191.0 | 2.92E-42 | 60 |
| 68 | H_4 | H | 4 | 0.839 | 0.813 | 0.014 | 0.030 | 0.009 | 0.009 | 0.000 | 0.119 | 0.004 | 6663.9 | 130.8 | 3.50E-29 | 54 |
| 69 | LQ_4 | LQ | 4 | 0.753 | 0.739 | 0.022 | 0.031 | 0.013 | 0.006 | 0.000 | 0.100 | 0.003 | 6773.1 | 240.0 | 6.57E-53 | 27 |
| 70 | HQC_4 | HQC | 4 | 0.820 | 0.798 | 0.018 | 0.032 | 0.010 | 0.003 | 0.000 | 0.109 | 0.004 | 6637.8 | 104.7 | 1.59E-23 | 47 |
| 71 | LQP_4 | LQP | 4 | 0.798 | 0.779 | 0.020 | 0.033 | 0.014 | 0.006 | 0.000 | 0.106 | 0.002 | 6718.0 | 184.9 | 6.18E-41 | 56 |
| 72 | HQP_4 | HQP | 4 | 0.853 | 0.822 | 0.013 | 0.032 | 0.010 | 0.003 | 0.000 | 0.125 | 0.004 | 6625.6 | 92.5 | 7.27E-21 | 75 |
| 73 | L_4.5 | L | 4.5 | 0.730 | 0.719 | 0.024 | 0.030 | 0.011 | 0.003 | 0.000 | 0.109 | 0.003 | 6797.4 | 264.3 | 3.50E-58 | 18 |
| 74 | Q_4.5 | Q | 4.5 | 0.741 | 0.730 | 0.023 | 0.030 | 0.011 | 0.006 | 0.000 | 0.103 | 0.002 | 6778.0 | 244.9 | 5.73E-54 | 20 |
| 75 | P_4.5 | P | 4.5 | 0.786 | 0.766 | 0.020 | 0.034 | 0.014 | 0.006 | 0.000 | 0.116 | 0.002 | 6749.9 | 216.8 | 7.33E-48 | 58 |
| 76 | H_4.5 | H | 4.5 | 0.827 | 0.800 | 0.016 | 0.031 | 0.009 | 0.013 | 0.000 | 0.125 | 0.004 | 6693.1 | 160.0 | 1.59E-35 | 48 |
| 77 | LQ_4.5 | LQ | 4.5 | 0.747 | 0.733 | 0.023 | 0.031 | 0.012 | 0.006 | 0.000 | 0.103 | 0.003 | 6777.0 | 243.9 | 9.78E-54 | 24 |
| 78 | HQC_4.5 | HQC | 4.5 | 0.808 | 0.785 | 0.018 | 0.031 | 0.010 | 0.006 | 0.000 | 0.113 | 0.004 | 6663.9 | 130.8 | 3.41E-29 | 42 |
| 79 | LQP_4.5 | LQP | 4.5 | 0.789 | 0.772 | 0.020 | 0.032 | 0.013 | 0.006 | 0.000 | 0.109 | 0.004 | 6731.7 | 198.6 | 6.44E-44 | 54 |
| 80 | HQP_4.5 | HQP | 4.5 | 0.837 | 0.811 | 0.015 | 0.033 | 0.011 | 0.003 | 0.000 | 0.116 | 0.004 | 6642.9 | 109.8 | 1.24E-24 | 66 |
| 81 | L_5 | L | 5 | 0.730 | 0.718 | 0.024 | 0.030 | 0.011 | 0.003 | 0.000 | 0.109 | 0.003 | 6797.1 | 264.0 | 4.12E-58 | 17 |
| 82 | Q_5 | Q | 5 | 0.739 | 0.728 | 0.022 | 0.029 | 0.011 | 0.003 | 0.000 | 0.100 | 0.002 | 6782.4 | 249.3 | 6.55E-55 | 19 |
| 83 | P_5 | P | 5 | 0.782 | 0.763 | 0.019 | 0.033 | 0.012 | 0.006 | 0.000 | 0.113 | 0.002 | 6733.2 | 200.1 | 3.05E-44 | 49 |
| 84 | H_5 | H | 5 | 0.813 | 0.785 | 0.018 | 0.031 | 0.010 | 0.013 | 0.001 | 0.131 | 0.004 | 6722.3 | 189.2 | 7.29E-42 | 41 |
| 85 | LQ_5 | LQ | 5 | 0.744 | 0.730 | 0.023 | 0.031 | 0.012 | 0.006 | 0.000 | 0.109 | 0.003 | 6778.0 | 244.9 | 5.88E-54 | 21 |
| 86 | HQC_5 | HQC | 5 | 0.795 | 0.773 | 0.019 | 0.030 | 0.010 | 0.006 | 0.000 | 0.113 | 0.003 | 6699.5 | 166.4 | 6.60E-37 | 40 |
| 87 | LQP_5 | LQP | 5 | 0.783 | 0.767 | 0.022 | 0.032 | 0.013 | 0.006 | 0.000 | 0.113 | 0.003 | 6722.4 | 189.3 | 7.01E-42 | 44 |
| 88 | HQP_5 | HQP | 5 | 0.827 | 0.801 | 0.015 | 0.032 | 0.010 | 0.003 | 0.000 | 0.116 | 0.002 | 6670.4 | 137.3 | 1.33E-30 | 61 |
| 89 | L_5.5 | L | 5.5 | 0.729 | 0.718 | 0.024 | 0.030 | 0.011 | 0.003 | 0.000 | 0.109 | 0.003 | 6797.0 | 263.9 | 4.44E-58 | 16 |
| 90 | Q_5.5 | Q | 5.5 | 0.736 | 0.727 | 0.023 | 0.029 | 0.011 | 0.003 | 0.000 | 0.100 | 0.002 | 6783.8 | 250.7 | 3.20E-55 | 17 |
| 91 | P_5.5 | P | 5.5 | 0.774 | 0.756 | 0.021 | 0.033 | 0.013 | 0.006 | 0.000 | 0.113 | 0.002 | 6745.1 | 212.0 | 8.16E-47 | 45 |
| 92 | H_5.5 | H | 5.5 | 0.797 | 0.773 | 0.020 | 0.031 | 0.010 | 0.009 | 0.000 | 0.131 | 0.005 | 6734.2 | 201.1 | 1.88E-44 | 30 |
| 93 | LQ_5.5 | LQ | 5.5 | 0.742 | 0.729 | 0.023 | 0.030 | 0.012 | 0.003 | 0.000 | 0.100 | 0.003 | 6780.9 | 247.8 | 1.34E-54 | 20 |
| 94 | HQC_5.5 | HQC | 5.5 | 0.784 | 0.764 | 0.019 | 0.029 | 0.010 | 0.006 | 0.000 | 0.106 | 0.003 | 6711.2 | 178.1 | 1.87E-39 | 32 |
| 95 | LQP_5.5 | LQP | 5.5 | 0.780 | 0.762 | 0.021 | 0.033 | 0.014 | 0.003 | 0.000 | 0.113 | 0.003 | 6719.1 | 186.0 | 3.61E-41 | 39 |
| 96 | HQP_5.5 | HQP | 5.5 | 0.817 | 0.794 | 0.016 | 0.032 | 0.011 | 0.003 | 0.000 | 0.119 | 0.003 | 6684.2 | 151.1 | 1.38E-33 | 56 |
| 97 | L_6 | L | 6 | 0.728 | 0.717 | 0.024 | 0.030 | 0.011 | 0.003 | 0.000 | 0.116 | 0.002 | 6799.1 | 266.0 | 1.51E-58 | 16 |
| 98 | Q_6 | Q | 6 | 0.735 | 0.726 | 0.023 | 0.028 | 0.010 | 0.003 | 0.000 | 0.100 | 0.002 | 6782.4 | 249.3 | 6.34E-55 | 15 |
| 99 | P_6 | P | 6 | 0.762 | 0.746 | 0.021 | 0.033 | 0.013 | 0.006 | 0.000 | 0.109 | 0.003 | 6747.3 | 214.2 | 2.68E-47 | 37 |
| 100 | H_6 | H | 6 | 0.786 | 0.762 | 0.023 | 0.030 | 0.011 | 0.009 | 0.000 | 0.131 | 0.006 | 6766.1 | 233.0 | 2.17E-51 | 31 |
| 101 | LQ_6 | LQ | 6 | 0.739 | 0.728 | 0.023 | 0.030 | 0.012 | 0.003 | 0.000 | 0.103 | 0.002 | 6785.3 | 252.3 | 1.47E-55 | 19 |
| 102 | HQC_6 | HQC | 6 | 0.777 | 0.759 | 0.019 | 0.028 | 0.009 | 0.006 | 0.000 | 0.109 | 0.003 | 6722.0 | 189.0 | 8.18E-42 | 29 |
| 103 | LQP_6 | LQP | 6 | 0.771 | 0.757 | 0.023 | 0.033 | 0.014 | 0.003 | 0.000 | 0.103 | 0.003 | 6739.5 | 206.4 | 1.34E-45 | 39 |
| 104 | HQP_6 | HQP | 6 | 0.812 | 0.783 | 0.017 | 0.033 | 0.011 | 0.006 | 0.000 | 0.109 | 0.004 | 6673.1 | 140.0 | 3.44E-31 | 47 |
| 105 | L_6.5 | L | 6.5 | 0.727 | 0.716 | 0.024 | 0.030 | 0.011 | 0.003 | 0.000 | 0.113 | 0.002 | 6801.7 | 268.6 | 4.19E-59 | 16 |
| 106 | Q_6.5 | Q | 6.5 | 0.734 | 0.725 | 0.022 | 0.028 | 0.010 | 0.003 | 0.000 | 0.103 | 0.002 | 6787.3 | 254.2 | 5.47E-56 | 16 |
| 107 | P_6.5 | P | 6.5 | 0.761 | 0.740 | 0.022 | 0.034 | 0.013 | 0.006 | 0.000 | 0.106 | 0.003 | 6744.7 | 211.6 | 1.00E-46 | 36 |
| 108 | H_6.5 | H | 6.5 | 0.776 | 0.753 | 0.021 | 0.029 | 0.011 | 0.006 | 0.000 | 0.134 | 0.004 | 6779.4 | 246.3 | 2.88E-54 | 26 |
| 109 | LQ_6.5 | LQ | 6.5 | 0.738 | 0.727 | 0.023 | 0.029 | 0.011 | 0.003 | 0.000 | 0.106 | 0.002 | 6787.0 | 253.9 | 6.51E-56 | 18 |
| 110 | HQC_6.5 | HQC | 6.5 | 0.770 | 0.753 | 0.018 | 0.028 | 0.009 | 0.006 | 0.000 | 0.103 | 0.002 | 6736.7 | 203.6 | 5.32E-45 | 28 |
| 111 | LQP_6.5 | LQP | 6.5 | 0.766 | 0.752 | 0.023 | 0.033 | 0.014 | 0.006 | 0.000 | 0.103 | 0.003 | 6737.1 | 204.0 | 4.38E-45 | 32 |
| 112 | HQP_6.5 | HQP | 6.5 | 0.800 | 0.774 | 0.018 | 0.033 | 0.012 | 0.003 | 0.000 | 0.119 | 0.003 | 6699.7 | 166.6 | 5.75E-37 | 45 |
| 113 | L_7 | L | 7 | 0.726 | 0.715 | 0.023 | 0.030 | 0.010 | 0.006 | 0.000 | 0.116 | 0.002 | 6802.1 | 269.0 | 3.36E-59 | 15 |
| 114 | Q_7 | Q | 7 | 0.733 | 0.724 | 0.022 | 0.028 | 0.010 | 0.003 | 0.000 | 0.106 | 0.003 | 6788.0 | 254.9 | 3.98E-56 | 15 |
| 115 | P_7 | P | 7 | 0.751 | 0.734 | 0.024 | 0.033 | 0.013 | 0.003 | 0.000 | 0.094 | 0.003 | 6758.0 | 224.9 | 1.27E-49 | 31 |
| 116 | H_7 | H | 7 | 0.762 | 0.748 | 0.020 | 0.027 | 0.009 | 0.006 | 0.000 | 0.134 | 0.004 | 6798.8 | 265.7 | 1.81E-58 | 26 |
| 117 | LQ_7 | LQ | 7 | 0.737 | 0.726 | 0.022 | 0.029 | 0.011 | 0.003 | 0.000 | 0.103 | 0.002 | 6792.4 | 259.3 | 4.39E-57 | 19 |
| 118 | HQC_7 | HQC | 7 | 0.766 | 0.750 | 0.018 | 0.027 | 0.008 | 0.006 | 0.000 | 0.103 | 0.002 | 6741.3 | 208.2 | 5.40E-46 | 24 |
| 119 | LQP_7 | LQP | 7 | 0.758 | 0.747 | 0.024 | 0.032 | 0.014 | 0.006 | 0.000 | 0.097 | 0.003 | 6749.8 | 216.7 | 7.90E-48 | 30 |
| 120 | HQP_7 | HQP | 7 | 0.789 | 0.768 | 0.018 | 0.032 | 0.012 | 0.009 | 0.000 | 0.116 | 0.003 | 6710.6 | 177.5 | 2.51E-39 | 39 |
| 121 | L_7.5 | L | 7.5 | 0.725 | 0.714 | 0.023 | 0.030 | 0.010 | 0.006 | 0.000 | 0.113 | 0.003 | 6805.2 | 272.1 | 7.23E-60 | 15 |
| 122 | Q_7.5 | Q | 7.5 | 0.732 | 0.723 | 0.022 | 0.028 | 0.010 | 0.006 | 0.000 | 0.106 | 0.003 | 6790.9 | 257.8 | 8.96E-57 | 15 |
| 123 | P_7.5 | P | 7.5 | 0.745 | 0.728 | 0.024 | 0.034 | 0.013 | 0.003 | 0.000 | 0.103 | 0.003 | 6762.7 | 229.6 | 1.22E-50 | 27 |
| 124 | H_7.5 | H | 7.5 | 0.759 | 0.744 | 0.020 | 0.028 | 0.010 | 0.006 | 0.000 | 0.134 | 0.004 | 6801.4 | 268.3 | 4.75E-59 | 21 |
| 125 | LQ_7.5 | LQ | 7.5 | 0.736 | 0.725 | 0.022 | 0.029 | 0.011 | 0.003 | 0.000 | 0.100 | 0.002 | 6792.7 | 259.6 | 3.73E-57 | 18 |
| 126 | HQC_7.5 | HQC | 7.5 | 0.761 | 0.747 | 0.018 | 0.026 | 0.008 | 0.006 | 0.000 | 0.103 | 0.002 | 6751.5 | 218.4 | 3.23E-48 | 23 |
| 127 | LQP_7.5 | LQP | 7.5 | 0.758 | 0.743 | 0.024 | 0.032 | 0.014 | 0.006 | 0.000 | 0.103 | 0.003 | 6745.7 | 212.6 | 6.06E-47 | 27 |
| 128 | HQP_7.5 | HQP | 7.5 | 0.785 | 0.763 | 0.018 | 0.031 | 0.011 | 0.006 | 0.000 | 0.119 | 0.003 | 6711.5 | 178.5 | 1.56E-39 | 34 |
| 129 | L_8 | L | 8 | 0.724 | 0.713 | 0.023 | 0.030 | 0.010 | 0.006 | 0.000 | 0.113 | 0.003 | 6808.4 | 275.3 | 1.47E-60 | 15 |
| 130 | Q_8 | Q | 8 | 0.731 | 0.722 | 0.022 | 0.028 | 0.010 | 0.006 | 0.000 | 0.106 | 0.003 | 6791.8 | 258.7 | 5.77E-57 | 14 |
| 131 | P_8 | P | 8 | 0.738 | 0.723 | 0.025 | 0.033 | 0.013 | 0.003 | 0.000 | 0.106 | 0.003 | 6774.3 | 241.2 | 3.66E-53 | 26 |
| 132 | H_8 | H | 8 | 0.756 | 0.742 | 0.020 | 0.027 | 0.010 | 0.006 | 0.000 | 0.125 | 0.005 | 6810.6 | 277.5 | 4.89E-61 | 19 |
| 133 | LQ_8 | LQ | 8 | 0.735 | 0.724 | 0.022 | 0.029 | 0.011 | 0.003 | 0.000 | 0.100 | 0.002 | 6793.6 | 260.5 | 2.40E-57 | 17 |
| 134 | HQC_8 | HQC | 8 | 0.758 | 0.744 | 0.018 | 0.026 | 0.008 | 0.006 | 0.000 | 0.106 | 0.002 | 6765.0 | 231.9 | 3.91E-51 | 25 |
| 135 | LQP_8 | LQP | 8 | 0.753 | 0.739 | 0.025 | 0.032 | 0.014 | 0.006 | 0.000 | 0.103 | 0.002 | 6754.5 | 221.4 | 7.48E-49 | 24 |
| 136 | HQP_8 | HQP | 8 | 0.777 | 0.757 | 0.020 | 0.031 | 0.012 | 0.006 | 0.000 | 0.119 | 0.003 | 6732.7 | 199.6 | 3.90E-44 | 35 |
| 137 | L_8.5 | L | 8.5 | 0.722 | 0.711 | 0.023 | 0.030 | 0.010 | 0.006 | 0.000 | 0.113 | 0.003 | 6809.4 | 276.3 | 8.79E-61 | 14 |
| 138 | Q_8.5 | Q | 8.5 | 0.729 | 0.721 | 0.022 | 0.028 | 0.009 | 0.006 | 0.000 | 0.109 | 0.002 | 6795.2 | 262.1 | 1.09E-57 | 14 |
| 139 | P_8.5 | P | 8.5 | 0.733 | 0.717 | 0.026 | 0.035 | 0.014 | 0.003 | 0.000 | 0.113 | 0.003 | 6783.0 | 249.9 | 4.86E-55 | 24 |
| 140 | H_8.5 | H | 8.5 | 0.753 | 0.740 | 0.021 | 0.028 | 0.010 | 0.006 | 0.000 | 0.125 | 0.005 | 6825.2 | 292.1 | 3.33E-64 | 19 |
| 141 | LQ_8.5 | LQ | 8.5 | 0.733 | 0.722 | 0.023 | 0.029 | 0.011 | 0.003 | 0.000 | 0.097 | 0.002 | 6798.8 | 265.7 | 1.77E-58 | 18 |
| 142 | HQC_8.5 | HQC | 8.5 | 0.755 | 0.742 | 0.018 | 0.026 | 0.008 | 0.006 | 0.000 | 0.106 | 0.002 | 6765.7 | 232.6 | 2.71E-51 | 22 |
| 143 | LQP_8.5 | LQP | 8.5 | 0.749 | 0.736 | 0.025 | 0.032 | 0.014 | 0.006 | 0.000 | 0.113 | 0.002 | 6765.3 | 232.2 | 3.40E-51 | 25 |
| 144 | HQP_8.5 | HQP | 8.5 | 0.773 | 0.752 | 0.020 | 0.032 | 0.012 | 0.006 | 0.000 | 0.122 | 0.003 | 6732.3 | 199.2 | 4.81E-44 | 30 |
| 145 | L_9 | L | 9 | 0.721 | 0.710 | 0.023 | 0.030 | 0.010 | 0.009 | 0.001 | 0.116 | 0.003 | 6812.9 | 279.8 | 1.51E-61 | 14 |
| 146 | Q_9 | Q | 9 | 0.728 | 0.720 | 0.022 | 0.028 | 0.009 | 0.006 | 0.000 | 0.113 | 0.002 | 6798.6 | 265.5 | 1.97E-58 | 14 |
| 147 | P_9 | P | 9 | 0.728 | 0.714 | 0.026 | 0.034 | 0.013 | 0.003 | 0.000 | 0.113 | 0.003 | 6791.3 | 258.2 | 7.67E-57 | 23 |
| 148 | H_9 | H | 9 | 0.752 | 0.737 | 0.020 | 0.028 | 0.010 | 0.006 | 0.000 | 0.125 | 0.005 | 6833.1 | 300.0 | 6.15E-66 | 17 |
| 149 | LQ_9 | LQ | 9 | 0.732 | 0.721 | 0.022 | 0.029 | 0.011 | 0.003 | 0.000 | 0.097 | 0.002 | 6800.0 | 266.9 | 9.53E-59 | 17 |
| 150 | HQC_9 | HQC | 9 | 0.752 | 0.739 | 0.018 | 0.026 | 0.008 | 0.006 | 0.000 | 0.109 | 0.002 | 6768.5 | 235.4 | 6.77E-52 | 20 |
| 151 | LQP_9 | LQP | 9 | 0.746 | 0.734 | 0.025 | 0.032 | 0.014 | 0.009 | 0.001 | 0.103 | 0.002 | 6766.0 | 232.9 | 2.30E-51 | 22 |
| 152 | HQP_9 | HQP | 9 | 0.768 | 0.747 | 0.021 | 0.031 | 0.012 | 0.006 | 0.000 | 0.116 | 0.003 | 6735.6 | 202.5 | 9.14E-45 | 26 |
| 153 | L_10 | L | 10 | 0.718 | 0.707 | 0.023 | 0.030 | 0.009 | 0.013 | 0.002 | 0.116 | 0.003 | 6820.7 | 287.6 | 3.04E-63 | 14 |
| 154 | Q_10 | Q | 10 | 0.725 | 0.717 | 0.022 | 0.028 | 0.009 | 0.013 | 0.002 | 0.106 | 0.003 | 6803.7 | 270.6 | 1.56E-59 | 13 |
| 155 | P_10 | P | 10 | 0.723 | 0.709 | 0.026 | 0.033 | 0.013 | 0.003 | 0.000 | 0.116 | 0.003 | 6798.4 | 265.3 | 2.20E-58 | 21 |
| 156 | H_10 | H | 10 | 0.745 | 0.732 | 0.019 | 0.028 | 0.009 | 0.006 | 0.000 | 0.122 | 0.004 | 6858.4 | 325.3 | 2.02E-71 | 15 |
| 157 | LQ_10 | LQ | 10 | 0.729 | 0.719 | 0.022 | 0.029 | 0.010 | 0.006 | 0.000 | 0.109 | 0.002 | 6805.6 | 272.5 | 5.98E-60 | 16 |
| 158 | HQC_10 | HQC | 10 | 0.747 | 0.733 | 0.019 | 0.027 | 0.008 | 0.013 | 0.002 | 0.113 | 0.002 | 6774.4 | 241.3 | 3.51E-53 | 17 |
| 159 | LQP_10 | LQP | 10 | 0.743 | 0.730 | 0.025 | 0.032 | 0.014 | 0.009 | 0.001 | 0.106 | 0.002 | 6772.4 | 239.3 | 9.68E-53 | 21 |
| 160 | HQP_10 | HQP | 10 | 0.760 | 0.741 | 0.022 | 0.032 | 0.013 | 0.006 | 0.000 | 0.116 | 0.003 | 6754.9 | 221.8 | 6.12E-49 | 26 |
| 161 | L_10.5 | L | 10.5 | 0.717 | 0.705 | 0.022 | 0.030 | 0.009 | 0.013 | 0.002 | 0.113 | 0.003 | 6824.8 | 291.7 | 4.03E-64 | 14 |
| 162 | Q_10.5 | Q | 10.5 | 0.724 | 0.716 | 0.023 | 0.028 | 0.009 | 0.013 | 0.002 | 0.113 | 0.003 | 6807.0 | 273.9 | 2.96E-60 | 13 |
| 163 | P_10.5 | P | 10.5 | 0.721 | 0.707 | 0.027 | 0.033 | 0.013 | 0.003 | 0.000 | 0.113 | 0.003 | 6798.9 | 265.8 | 1.70E-58 | 19 |
| 164 | H_10.5 | H | 10.5 | 0.743 | 0.730 | 0.020 | 0.028 | 0.009 | 0.006 | 0.000 | 0.119 | 0.004 | 6867.2 | 334.1 | 2.46E-73 | 12 |
| 165 | LQ_10.5 | LQ | 10.5 | 0.728 | 0.717 | 0.022 | 0.029 | 0.010 | 0.009 | 0.001 | 0.109 | 0.002 | 6809.4 | 276.3 | 8.67E-61 | 16 |
| 166 | HQC_10.5 | HQC | 10.5 | 0.744 | 0.730 | 0.020 | 0.027 | 0.008 | 0.013 | 0.002 | 0.119 | 0.003 | 6781.2 | 248.1 | 1.16E-54 | 17 |
| 167 | LQP_10.5 | LQP | 10.5 | 0.742 | 0.729 | 0.025 | 0.032 | 0.014 | 0.006 | 0.000 | 0.103 | 0.002 | 6774.1 | 241.0 | 4.02E-53 | 20 |
| 168 | HQP_10.5 | HQP | 10.5 | 0.758 | 0.738 | 0.023 | 0.032 | 0.013 | 0.006 | 0.000 | 0.116 | 0.003 | 6755.2 | 222.1 | 5.15E-49 | 23 |
| 169 | L_11 | L | 11 | 0.715 | 0.703 | 0.022 | 0.030 | 0.009 | 0.013 | 0.002 | 0.109 | 0.004 | 6829.4 | 296.3 | 4.03E-65 | 14 |
| 170 | Q_11 | Q | 11 | 0.722 | 0.714 | 0.023 | 0.028 | 0.009 | 0.013 | 0.002 | 0.106 | 0.003 | 6810.5 | 277.4 | 5.10E-61 | 13 |
| 171 | P_11 | P | 11 | 0.718 | 0.704 | 0.027 | 0.033 | 0.013 | 0.003 | 0.000 | 0.113 | 0.003 | 6806.9 | 273.8 | 3.02E-60 | 20 |
| 172 | H_11 | H | 11 | 0.741 | 0.727 | 0.020 | 0.028 | 0.009 | 0.006 | 0.000 | 0.113 | 0.003 | 6882.6 | 349.5 | 1.15E-76 | 12 |
| 173 | LQ_11 | LQ | 11 | 0.726 | 0.716 | 0.023 | 0.029 | 0.010 | 0.013 | 0.002 | 0.113 | 0.002 | 6810.6 | 277.5 | 4.74E-61 | 15 |
| 174 | HQC_11 | HQC | 11 | 0.742 | 0.726 | 0.021 | 0.028 | 0.008 | 0.013 | 0.002 | 0.113 | 0.003 | 6787.7 | 254.6 | 4.53E-56 | 17 |
| 175 | LQP_11 | LQP | 11 | 0.740 | 0.727 | 0.025 | 0.032 | 0.014 | 0.009 | 0.001 | 0.100 | 0.002 | 6779.5 | 246.4 | 2.73E-54 | 20 |
| 176 | HQP_11 | HQP | 11 | 0.755 | 0.735 | 0.023 | 0.032 | 0.013 | 0.006 | 0.000 | 0.116 | 0.002 | 6769.6 | 236.5 | 3.92E-52 | 26 |
| 177 | L_11.5 | L | 11.5 | 0.713 | 0.702 | 0.022 | 0.030 | 0.009 | 0.009 | 0.001 | 0.109 | 0.004 | 6833.8 | 300.7 | 4.51E-66 | 14 |
| 178 | Q_11.5 | Q | 11.5 | 0.721 | 0.712 | 0.023 | 0.029 | 0.009 | 0.013 | 0.002 | 0.106 | 0.003 | 6814.4 | 281.3 | 7.33E-62 | 13 |
| 179 | P_11.5 | P | 11.5 | 0.715 | 0.701 | 0.027 | 0.034 | 0.012 | 0.003 | 0.000 | 0.119 | 0.002 | 6812.8 | 279.7 | 1.62E-61 | 20 |
| 180 | H_11.5 | H | 11.5 | 0.739 | 0.724 | 0.020 | 0.028 | 0.009 | 0.003 | 0.000 | 0.109 | 0.002 | 6895.6 | 362.5 | 1.71E-79 | 11 |
| 181 | LQ_11.5 | LQ | 11.5 | 0.725 | 0.714 | 0.023 | 0.029 | 0.010 | 0.013 | 0.002 | 0.106 | 0.003 | 6816.4 | 283.3 | 2.65E-62 | 16 |
| 182 | HQC_11.5 | HQC | 11.5 | 0.739 | 0.723 | 0.021 | 0.028 | 0.008 | 0.009 | 0.001 | 0.106 | 0.002 | 6794.8 | 261.7 | 1.33E-57 | 17 |
| 183 | LQP_11.5 | LQP | 11.5 | 0.739 | 0.725 | 0.025 | 0.031 | 0.014 | 0.009 | 0.001 | 0.100 | 0.002 | 6783.5 | 250.4 | 3.68E-55 | 20 |
| 184 | HQP_11.5 | HQP | 11.5 | 0.752 | 0.731 | 0.024 | 0.032 | 0.013 | 0.009 | 0.001 | 0.109 | 0.003 | 6777.2 | 244.1 | 8.66E-54 | 26 |
| 185 | L_12 | L | 12 | 0.711 | 0.700 | 0.023 | 0.030 | 0.009 | 0.009 | 0.001 | 0.109 | 0.004 | 6835.7 | 302.6 | 1.73E-66 | 13 |
| 186 | Q_12 | Q | 12 | 0.719 | 0.711 | 0.023 | 0.029 | 0.009 | 0.013 | 0.002 | 0.109 | 0.002 | 6815.8 | 282.7 | 3.67E-62 | 12 |
| 187 | P_12 | P | 12 | 0.713 | 0.699 | 0.027 | 0.034 | 0.012 | 0.003 | 0.000 | 0.116 | 0.002 | 6814.1 | 281.0 | 8.45E-62 | 18 |
| 188 | H_12 | H | 12 | 0.737 | 0.716 | 0.020 | 0.030 | 0.010 | 0.003 | 0.000 | 0.106 | 0.003 | 6916.7 | 383.6 | 4.33E-84 | 13 |
| 189 | LQ_12 | LQ | 12 | 0.723 | 0.713 | 0.023 | 0.029 | 0.010 | 0.013 | 0.002 | 0.106 | 0.003 | 6817.9 | 284.8 | 1.23E-62 | 15 |
| 190 | HQC_12 | HQC | 12 | 0.736 | 0.719 | 0.022 | 0.029 | 0.009 | 0.009 | 0.001 | 0.106 | 0.002 | 6799.6 | 266.5 | 1.21E-58 | 16 |
| 191 | LQP_12 | LQP | 12 | 0.737 | 0.722 | 0.026 | 0.032 | 0.014 | 0.009 | 0.001 | 0.103 | 0.002 | 6786.8 | 253.8 | 6.95E-56 | 19 |
| 192 | HQP_12 | HQP | 12 | 0.747 | 0.728 | 0.025 | 0.032 | 0.013 | 0.009 | 0.001 | 0.113 | 0.002 | 6778.1 | 245.0 | 5.65E-54 | 22 |

**Suppl. Table 2: Percent contribution and permutation importance of MVS base selected environmental variables.**

| Variables | Percent contribution | Permutation importance |
| --- | --- | --- |
| bio8 | 30.4 | 18.1 |
| bio13 | 17.3 | 4.1 |
| Forest_land | 16.6 | 17.8 |
| Soil_Clay_content | 14.7 | 20.5 |
| Cult_L | 10.3 | 10.4 |
| Grass_land | 7.7 | 19.3 |
| bio5 | 3 | 9.8 |

**Suppl. Table 3: AUC test values for individual models by population.**

| Species | Training AUC | Test AUC | AUC Standard Deviation | AUC with only Cult_T | AUC with only Forest_Land | AUC with only Grass_Land | AUC with the only Soil_Clay_Content | AUC with only bio5 | AUC with only bio8 | AUC with only bio13 | Entropy |
| --- | --- | --- | --- | --- | --- | --- | --- | --- | --- | --- | --- |
| Abergelle | 0.9899 | 0.9927 | 0.0059 | 0.8587 | 0.9004 | 0.7054 | 0.9047 | 0.9866 | 0.9674 | 0.8246 | 8.132 |
| Adwa | 0.9956 | 0.992 | 0.0021 | 0.9112 | 0.9792 | 0.9349 | 0.5581 | 0.9634 | 0.9069 | 0.5 | 7.3826 |
| Ahferom | 0.9884 | 0.9858 | 0.0049 | 0.7331 | 0.9364 | 0.6905 | 0.5 | 0.8922 | 0.8237 | 0.8445 | 7.8064 |
| Degua_Tembien | 0.985 | 0.9764 | 0.0158 | 0.8996 | 0.9576 | 0.8982 | 0.5 | 0.945 | 0.9632 | 0.8205 | 7.2071 |
| Hawzen | 0.9831 | 0.9789 | 0.0072 | 0.8795 | 0.9696 | 0.5948 | 0.8717 | 0.916 | 0.9275 | 0.9126 | 7.9142 |
| Hintalo_Wajrat | 0.9969 | 0.9974 | 0.0007 | 0.7267 | 0.9671 | 0.8778 | 0.5431 | 0.9041 | 0.8266 | 0.6796 | 8.0264 |
| Kafta_Humera | 0.9977 | 0.9978 | 0.001 | 0.9292 | 0.6358 | 0.939 | 0.8963 | 0.9571 | 0.9896 | 0.5 | 6.9562 |
| Laelay_Michew | 0.9787 | 0.9693 | 0.007 | 0.7814 | 0.8888 | 0.7838 | 0.6048 | 0.9403 | 0.8359 | 0.6618 | 8.1866 |
| Ofla | 0.9981 | 0.9986 | 0.0006 | 0.7063 | 0.9718 | 0.9845 | 0.8702 | 0.9006 | 0.9042 | 0.7474 | 5.6601 |
| Raya_Azebo | 0.9918 | 0.9873 | 0.0032 | 0.605 | 0.7269 | 0.6013 | 0.5 | 0.9817 | 0.9075 | 0.8407 | 7.7637 |
| Tahtay_Adyabo | 0.9938 | 0.9923 | 0.0021 | 0.9439 | 0.5566 | 0.9456 | 0.7914 | 0.9516 | 0.8675 | 0.5 | 7.4495 |
| Tahtay_Maichew | 0.9916 | 0.9938 | 0.0031 | 0.7719 | 0.8575 | 0.7174 | 0.8838 | 0.9824 | 0.9796 | 0.5 | 8.8479 |
| Tahtay_Qoraro | 0.9981 | 0.9946 | 0.0036 | 0.8959 | 0.957 | 0.865 | 0.8067 | 0.9756 | 0.9633 | 0.9676 | 7.5442 |
| Tsegedie | 0.9962 | 0.9942 | 0.0024 | 0.9766 | 0.9454 | 0.9481 | 0.9102 | 0.8617 | 0.9184 | 0.874 | 5.9461 |
| Tselemti | 0.9972 | 0.9986 | 0.0003 | 0.9921 | 0.89 | 0.9681 | 0.8153 | 0.964 | 0.9442 | 0.5 | 8.2329 |
| Welkayt | 0.9879 | 0.9923 | 0.0029 | 0.7464 | 0.9063 | 0.7265 | 0.8544 | 0.5641 | 0.7515 | 0.9262 | 7.2789 |


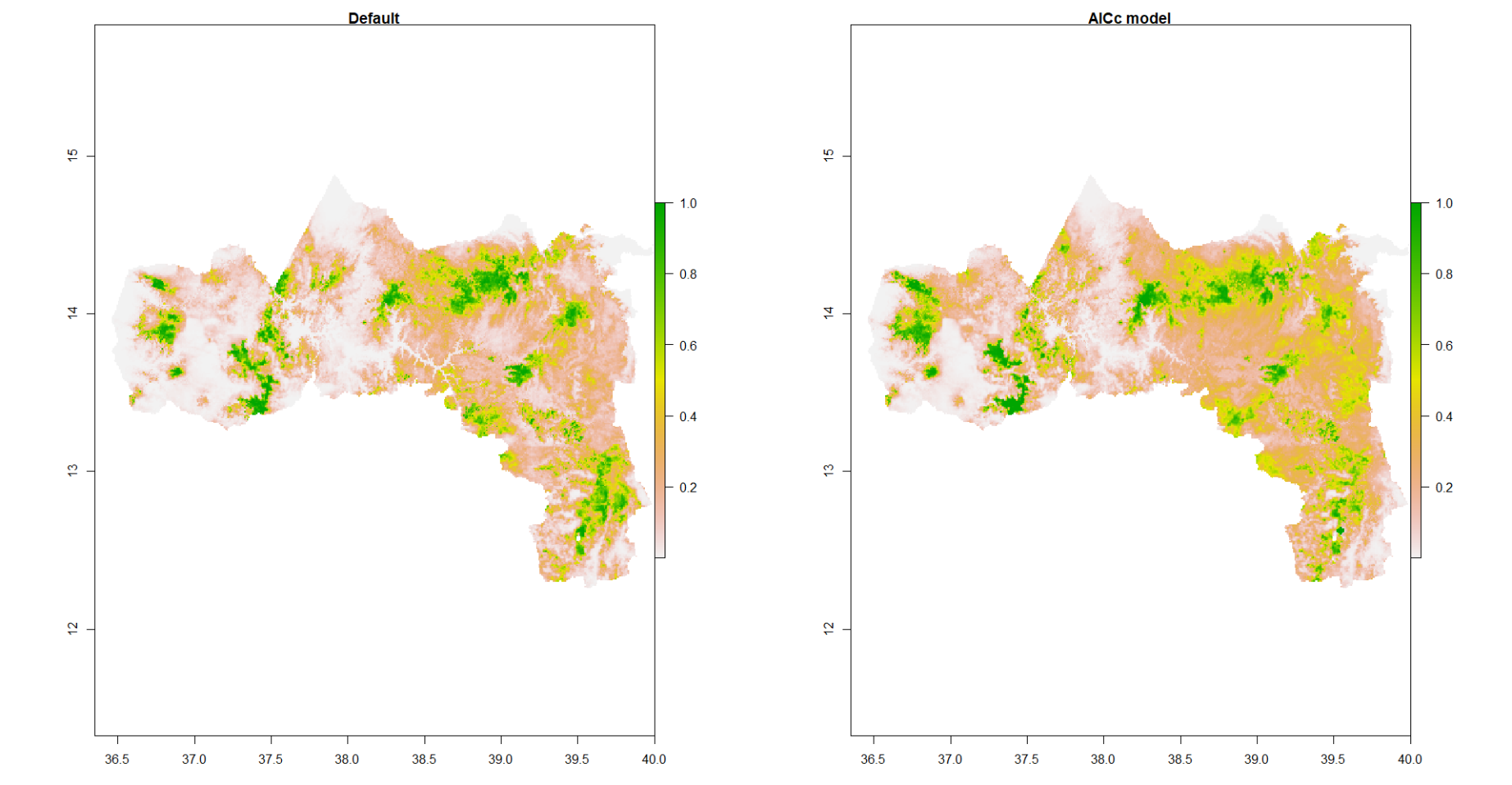


The habitat suitability increased

**Suppl. Figure 1: Suitability maps for Tigray chicken population using default settings provided by MaxEnt approach (Default), and with identified parameters (beta-multiplier =two & Hinge feature) using ENMeval (AICc model).**

**Suppl. Figure 2: Agglomerative coefficient value for hierarchical clustering using niche overlap.**

**Suppl. Figure 3: Agglomerative coefficient value for hierarchical clustering using Pearsons correlation.**
